# Quiescent cancer cells induced by high-density cultivation reveals cholesterol-mediated survival and lung metastatic traits

**DOI:** 10.1038/s41416-024-02861-x

**Published:** 2024-10-11

**Authors:** Xingyang Liu, Qinjie Min, Xinxin Cheng, Weimin Zhang, Qingnan Wu, Xu Chen, Mengzhu Lv, Siqi Liu, Huihui Zhao, Di Yang, Yidi Tai, Xiao Lei, Yan Wang, Qimin Zhan

**Affiliations:** 1 Key Laboratory of Carcinogenesis and Translational Research (Ministry of Education/Beijing), Laboratory of Molecular Oncology, Peking University Cancer Hospital & Institute, 100142 Beijing, China; 2 State Key Laboratory of Molecular Oncology, National Cancer Center/National Clinical Research Center for Cancer/Cancer Hospital, Chinese Academy of Medical Sciences and Peking Union Medical College, 100021 Beijing, China; 3 Peking University International Cancer Institute, 100191 Beijing, China; 4 Department of Biochemistry and Biophysics, School of Basic Medical Sciences, Peking University Health Science Center, 100191 Beijing, China; 5 Research Unit of Molecular Cancer Research, Chinese Academy of Medical Sciences, 100730 Beijing, China; 6 Soochow University Cancer Institute, Suzhou, 215000 China

**Keywords:** Cancer metabolism, Cell-cycle exit, Stress signalling, Cancer models

## Abstract

**Background:**

The metastatic cascade, a multifaceted and highly aggressive process, is the primary cause of mortality. The survival of quiescent cancer cells in circulatory system during metastasis is crucial, yet our comprehension is constrained by the absence of universally accepted quiescent cancer models.

**Method:**

We developed a quiescent cancer cell model using high-density cultivation. Based on the scRNA-seq analysis, IP-MS, metabolomics, mouse lung metastasis models, cholesterol assay, PLA and other molecular experiments, we explored the molecular mechanism. Immunofluorescence, atomic force microscope, FluidFM, and shear stress stimulation were used to analyze the cytoskeleton and membrane properties contributing to mechanical force resistance.

**Result:**

We established a quiescent cancer cell model induced by high-density cultivation. Single-cell RNA sequencing (scRNA-seq) analysis reveals that CDC25A plays a crucial role in the transition to quiescence, with its expression significantly elevated in the quiescent state. Depletion of CDC25A leads to an increased proliferative capacity, and reduced metastasis under high-density conditions. Mechanistically, upregulated CDC25A in quiescent cells enhances cholesterol metabolism via endosome pathways, leading to cell cycle arrest. This increase in cholesterol reinforces the cytoskeleton, alters membrane properties, and improves resistance to mechanical forces in circulatory system.

**Conclusion:**

CDC25A significantly increased the cholesterol metabolism through endosome pathway in quiescent cancer cells, leading to the significant changes in cytoskeleton and membrane properties so as to enhance the resistance of mechanical force in circulatory system, facilitating lung metastasis.

**Figure:**
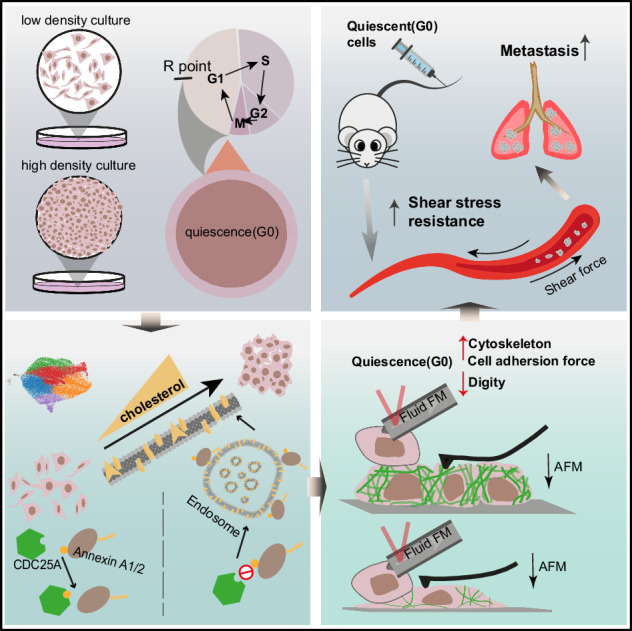
In high-density cultivation, quiescent cancer cells, up-regulate cholesterol metabolism by CDC25A through endosome pathway, enhancing the resistance to mechanical force in circulatory system, facilitating lung metastasis.

## Introduction

Metastasis, rather than the primary tumor growth, represents the most lethal outcome of cancer-related deaths [1]. The metastatic cascade which includes three main stages—local invasion, survival in the circulatory system, and distal colonization [1, 2]—are closely associated with the quiescent state. With the proliferation of cancer cells at the primary site, the crowded and confined environment induces the epithelial-mesenchymal transition, a quiescent state, leading to the acquisition of an invasive phenotype and the detachment from neighboring cell-cell contacts [3–5]. Disseminated cells in the circulatory system survive by maintaining quiescence under extensive stress of physical stress [6], chemotherapy, and other stress [7–9]. These biological processes show that the quiescent state of cancer cells participates in various metastatic steps [2, 10–14], and its regulation requires further investigation.

Numerous studies spanning several decades have provided evidence that the normal cells in the quiescent state can be driven during high-density cultivation [15, 16]. In contrast to normal cells, the quiescent cancer cells disseminated from primary solid tumor overcome contact inhibition. However, the lack of established models and markers hinders our understanding of the unique characteristics of quiescent cancer cells and their ability to survive in the circulatory system under constrained conditions. Further investigation is required to elucidate the distinct role of quiescent cancer cells induced by confined environments. Indeed, cancer cells in the circulatory system are in the quiescent state [17], indicating that high-density cultivation can empower quiescent cancer cells to resist environmental stress.

In our study, we induced the quiescent cancer cells using high-density cultivation to mimic the crowded environment inducing disseminated cancer cells. We verified the model through quiescent characteristics identified in stem cells and other quiescent cells, including low RNA content, low transcription due to inactive metabolism [15, 18], and decreased proliferative markers [19, 20]. Surprisingly, our scRNA-seq analysis revealed that a significant portion (approximately 60%) of cancer cells were capable of entering a quiescent state, and we filtered CDC25A, a member of the dual-specificity phosphatase family known as cell division cycle-25 (CDC25) [21, 22], role in entering quiescent state by increasing resistance of mechanical strength in the circulatory system through upregulating cholesterol levels. In summary, we established a quiescent model and demonstrated that whether CDC25A regulates cholesterol levels in quiescent cancer cells, thereby augmenting their resistance to chemotherapy and mechanical forces and facilitation of their metastatic potential.

## Results

### Establishment of quiescent cell model by high-density cultivation

**Fig. 1:**
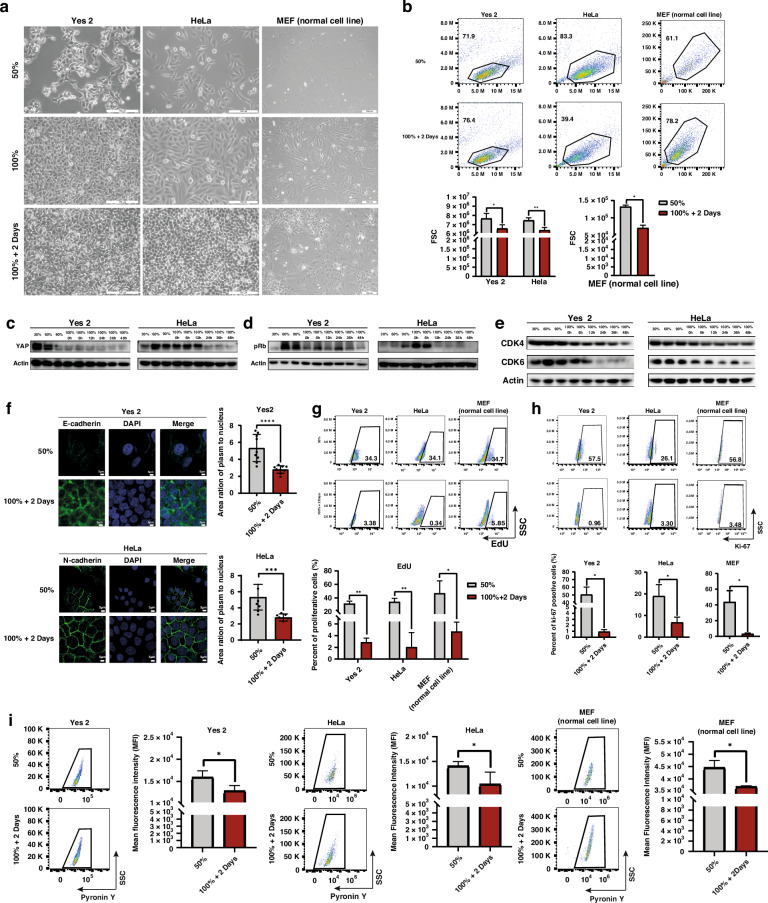

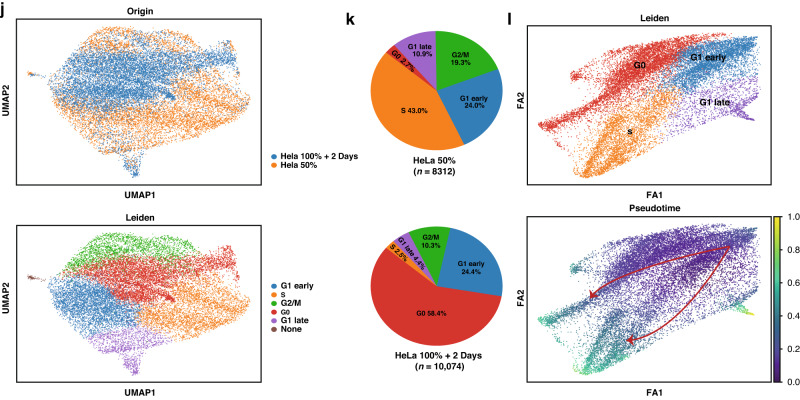
Establishment of cell quiescent model with high density and analysis by scRNA-seq. Gradients of cell densities were as follows: approximately 30% density (corresponding to the surface area of the cell cultivation dish), was chosen to represent sparse cells at the beginning of proliferation; approximately 50% density was used to represent cells in a proliferative state; 100% density indicated complete confluence of cells; and a density of 100%+2Days denoted cells that were cultured for an additional two days after reaching 100%, representing cells cultivated under high-density conditions. **a** Images of cell morphology in different cell densities. Cell line: Yes 2, HeLa, and MEF. **b** Flow cytometry plots of cell size in different cell densities. *p < 0.05, **p < 0.01. **c** Western Blot analysis of YAP expression in different cell densities. **d**, **e** Western Blot analysis of the expression of cell-cycle related protein. **f** Quantification of plasm to nucleus area ration by immunofluorescence analysis of E-cadherin positive Yes 2 cells, and N-cadherin positive HeLa cells *n* = 9. ***p < 0.001, ****p < 0.0001. **g** Flow cytometry plots of proliferative (EdU positive) cells. *p < 0.05, **p < 0.01. **h** Flow cytometry plots of proliferative (Ki-67 positive) cells. *p < 0.05. **i** RNA content assay by flow cytometry in different cell densities. Quantification by the mean fluorescent intensity (MFI) of pyronin Y. *p < 0.05. **j** Uniform manifold approximation and projection (UMAP) of single cell sequence, visualization of cell clusters of quiescent group-HeLa 100%+2Days, and proliferative group-HeLa 50%. **k** The proportion of clusters in **j**. **l** Pseudotime inference analysis of clusters determining the fate of cell cycle. The red lines represent the two cell trajectories of G_1_ early phase.

To mimic the confined and densely populated conditions inducing disseminated cancer cells from solid tumors, multiple gradients of cell density were selected. Building on current methods employed in various fields identifying quiescent cells [23–27], we verified our high-density quiescent cancer cell models. Our observations revealed significant changes in cell morphology (Fig. 1a) and a decrease in cell size in the 100%+2Days group when compared to the 30% and 100% density groups, both in HeLa and the normal cell line MEF cells (Fig. 1a, b). Additionally, the high-density culture did not induce excessive apoptosis (Supplementary Fig. 1a, b). The expression of YAP, a critical regulator of the Hippo pathway [27, 28], decreased as the cultivation density increasing (Fig. 1c). As previously reported, the Hippo pathway can be activated in high-density cultivation through cell polarity and junctional complexes, leading to induction of quiescence [23, 29–31]. Likewise, the levels of G_1_ phase-related proteins in the cell cycle, such as pRb and CDK4/6, decreased with increasing density (Fig. 1d, e). Additionally, the 100%+2Days group exhibited a noticeable decline in the cytoplasmic-to-nuclear area ratio of cells (Fig. 1f), and in the proliferative signal (5-ethynyl-2’-deoxyuridine (EdU) and Ki-67 antibody) compared with 50% density group (Fig. 1g, h). In addition, the RNA content assay showed that the RNA content in the 100%+2Days group was lower than that in the sparse groups following Pyronin Y staining (Fig. 1i). These results are consistent with the quiescent cells characteristics previously described, indicating that a substantial proportion of cancer cells in the high-density model entered the quiescent state. Therefore, the quiescent model induced by high-density cultivation was deemed suitable for further investigation.

### scRNA-seq analysis of the quiescent model reveals the regulation of quiescent entrance by CDC25A

**Fig. 2:**
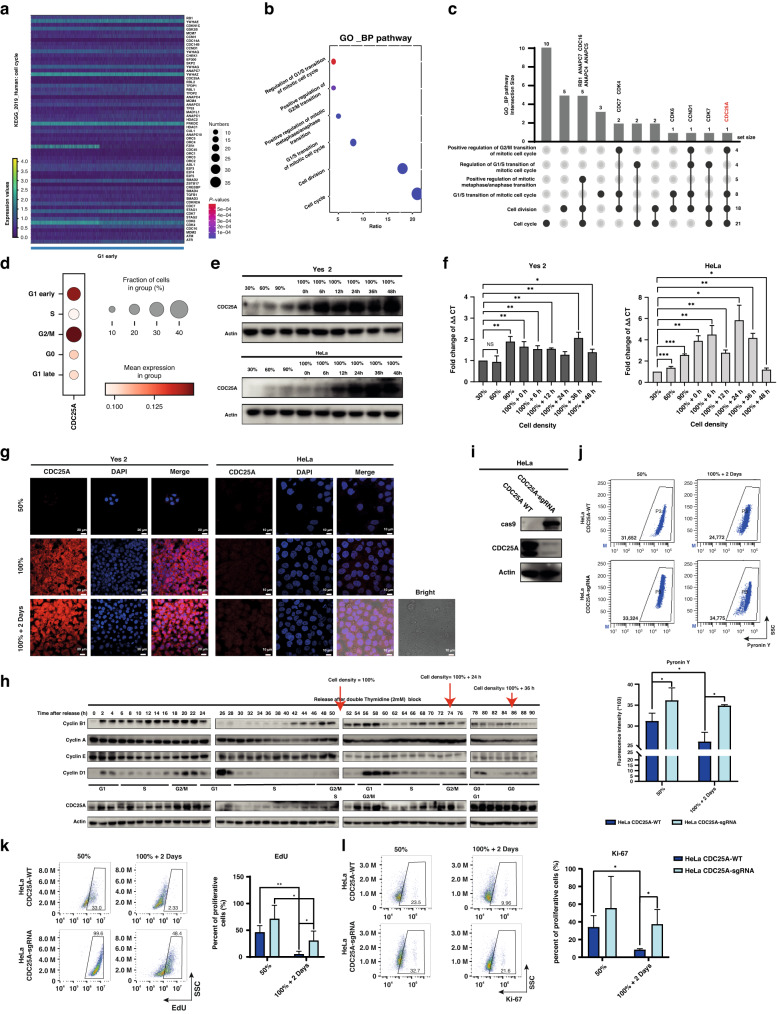
CDC25A plays a crucial role in entrance of cell quiescent state screened out by scRNA-seq analysis. **a** Gene expression of “cell cycle” pathways adjusted *p*-value of 0.02, enriched by KEGG in “G1 early”cluster. **b** Pathways related to cell cycle phases transition enriched by GO_BP analysis. **c**, UpSet plot of pathways in **b**. **d** The pattern of *CDC25A* expression in all clusters of single-cell UMAP plots. **e** Western Blot analysis of *CDC25A* expression in different cell densities. **f** RT-qPCR analysis of *CDC25A* expression in different cell densities. *p < 0.05, **p < 0.01. **g** Immunofluorescence analysis of CDC25A in different densities of tumor cells. **h** Western Blot analysis of CDC25A expression and cell-cycle-related markers after release from 2 mM double thymidine blocking cell cycle and releasing into mitosis. The cell density reached approximately 100% at 50 h after release. Red arrow: Time of release corresponding to the density reached at that point. **i** Western Blot analysis of the HeLa *CDC25A*-sgRNA cell line (knocked out *CDC25A*) after transfecting *CDC25A* sgRNA. **j**, RNA content assay by flow cytometry. Quantification by the mean fluorescent intensity (MFI). *p < 0.05. **k** Flow cytometry plots of proliferative (EdU positive) cells. *p < 0.05, **p < 0.01. **l**, Flow cytometry plots of proliferative (Ki-67 positive) cells. *p < 0.05.

To dissect quiescent cells under conditions of high heterogeneity in cancer cells, we clustered them and analyzed the highly differential genes induced by high-density cultivation at single-cell resolution. We obtained 18,386 high-quality HeLa cells, including 8312 cells from the HeLa 50% group and 10,074 cells from the HeLa 100%+2Days group. We then performed unsupervised clustering (Fig. 1j), distinguishing six clusters in different phases of cell cycle based on cell-cycle markers [32, 33]. The G_1_ phase compartment was bifurcated based on the G_1_ phase checkpoint, known as the “restriction point (R point) “, determining the trajectory of entrance into the quiescent state or irreversibly proceed with DNA replication and cell division (referred to as cluster “G1 early” or “G1 late”) (Fig. 1j) [34, 35]. The proportion of quiescent cells (“cluster G0”) is prominently increased as the major group after high-density culturing (Fig. 1k). After pseudotime inference analysis of quiescent induction, two trajectories of cell cycle were revealed, rooted by “G1 early”, of which “G1 early” enter into “G0” phase or irreversibly proceed with following cell cycle phases (Fig. 1l). To explore the molecular mechanisms of entering into quiescent state from cell cycle, the highly differential genes of cluster “G1 early” specifically enriched in “cell cycle” pathway (Fig. 2a). Then, the genes related to cell-cycle phase transition, enriched in “cell cycle” were analyzed (Fig. 2b). Notably, we observed that *CDC25A* was involved in most pathways of cell-cycle phase transition (Fig. 2c). Accordingly, *CDC25A* expression was highly induced in “G1 early” cluster (Fig. 2d). These findings suggested that a large proportion of cells could be induced into the quiescent state by high-density cultivation, with *CDC25A* potentially playing an important role in entering the quiescent state.

### *CDC25A* role in the determination of cellular entry into quiescent state

We next measured CDC25A expression increased at both protein (Fig. 2e, g) and transcriptional levels (Fig. 2f) in quiescent model, compared with the proliferative cell groups (30% and 50%). Previous research reported CDC25A role in the cell-cycle transition from G_2_ to M phase, thus, we examined the expression level of CDC25A throughout consecutive cell cycles until in the quiescent state. Our findings revealed oscillations of CDC25A expression during cell cycle transitions (Fig. 2c, d, h); however, a sustained high level was observed during prolonged high-density cultivation (after 100% + 24 h, Fig. 2h). These results suggest that CDC25A might plays a crucial role in the cell quiescent state.

To investigate the function of CDC25A in the quiescent state, we knocked out *CDC25A* in HeLa cell line using CRISPR/Cas9 (Fig. 2i). The proliferative capacity and RNA content of the *CDC25A*-sgRNA 100%+2Days group was greater than that of the *CDC25A*-WT cells (Fig. 2j-l). Together, the integrative analysis further supported the hypothesis that CDC25A played a crucial role in inducing the quiescent state. To validate the role of CDC25A, we established another quiescent model using starvation treatments and assessed several quiescent characteristics. Similarly, the expression levels of p-Rb and CDK4/6 decreased after 72 h and 96 h of starvation (Supplementary Fig. 2a, b). The cell size (Supplementary Fig. 2c) and proliferative capacity (Supplementary Fig. 2d, e) also decreased. Consequently, a significant number of cells entered the quiescent state following starvation. Consistent with the high-density model, we also observed an increase in CDC25A expression (Supplementary Fig. 2f), further confirming the function of CDC25A in quiescent state.

### CDC25A promotion of the malignant progression in quiescent tumor cells

**Fig. 3:**
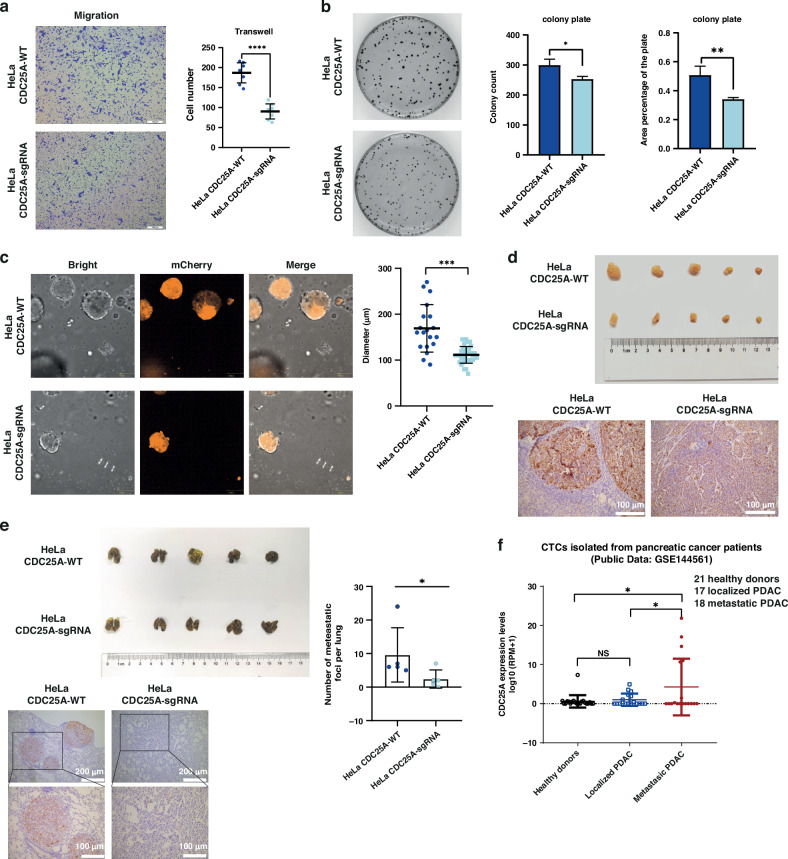
CDC25A of quiescent cells promotes the malignant progression of tumor. **a** Images of migrated cells after 24 h in transwell insert (left). Quantification (right) of migrated cells of HeLa *CDC25A*-WT cell line (*n* = 7) and HeLa *CDC25A*-sgRNA cell line (*n* = 8). ****p < 0.0001. **b** Colony formation assays (left). Quantification of colony count (middle) and colony area (right) of the colony. **c** Images and quantified diameter of sphere formation assay. ***p < 0.001. **d** Images of xenografts formation (*n* = 5 mice, Upper). Immunohistochemistry assays of Ki-67 positive cells in xenografts tumor (Lower). *p < 0.05. **e** Assessment of lung metastasis capacity via tail vein injection (*n* = 5 mice). Immunohistochemistry assays of Ki-67 positive cells in lung tissue. **f** The CDC25A expression levels of CTCs isolated from pancreatic cancer patients of published data (GSE144561). NS no significant. *p < 0.05.

In order to ascertain the oncogenic role of CDC25A, we conducted a survival analysis using the GEPIA database. The analysis revealed a statistically significant decrease in disease free survival (DFS) among individuals in the high-CDC25A group (log rank *p* = 0). Furthermore, CDC25A upregulation has been consistently associated with unfavorable prognoses across various cancer types [36, 37]. Subsequently, we assessed the malignant characteristics of the *CDC25A*-sgRNA 100%+2Days and *CDC25A*-WT 100%+2Days group, both in vitro and in vivo. We observed that the migratory ability of *CDC25A*-sgRNA cells was weaker than that of *CDC25A*-WT cells in the transwell system (Fig. 3a). Furthermore, the colony formation assay revealed a decrease in the capacity for colony formation and proliferation following CDC25A downregulation (Fig. 3b), consistent with the results of sphere formation assay (Fig. 3c). Additionally, *CDC25A* depletion inhibited the growth of xenografts (Fig. 3d) and the capacity of lung metastasis (Fig. 3e) in BALB/c-nu mice. Notably, the proliferative ability was enhanced following the deletion of *CDC25A* (Fig. 2k, l), thereby excluding the influence of the cell-cycle-promoting function of CDC25A on xenografts and lung metastasis (Fig. 3d, e). The quiescent *CDC25A*-WT cells injected into circulatory system through tail vein could be exposed to mechanical force stress directly in circulatory system, revealing a higher survival rate and a greater potential for lung metastasis compared with *CDC25A*-sgRNA cells. In addition, Circulating Tumor Cells (CTCs) isolated from metastatic pancreatic ductal adenocarcinoma (PDAC) patients showed significantly increased CDC25A expression compared to untreated localized PDAC patients (*n* = 17) (Fig. 3f), suggesting that upregulated CDC25A in CTCs may promote tumor metastasis in vivo. Collectively, it is suggested that the additional function of CDC25A in the quiescent state can contribute to the malignant progression by enhancing the resistance of mechanical force in the circulatory system.

### Up-regulation of cholesterol metabolism by CDC25A in quiescent state

**Fig. 4:**
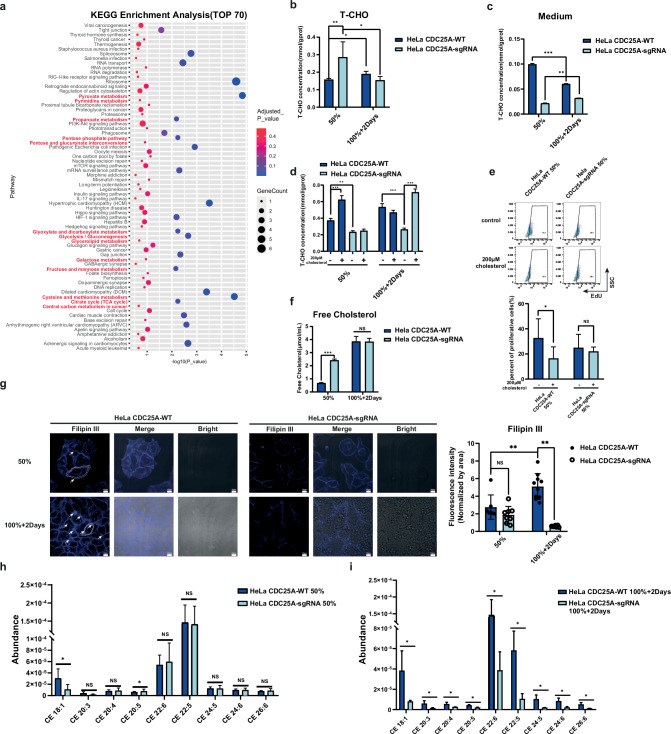
Cholesterol metabolism is upregulated by CDC25A in quiescent state. **a** KEGG enrichment analysis of the protein correlated with CDC25A, detect by immunoprecipitation-coupled mass spectrometry (IP-MS) and showing top70 upregulated pathways by adjusted p-value. The pathways related to metabolism are marked in red. **b**, **c** Total cholesterol assay in cells and in the culture medium. *p < 0.05, **p < 0.01, ***p < 0.001. **d** Total cholesterol assay in cells, cultured with additional 200 μM cholesterol. **p < 0.01, ***p < 0.001. **e** Flow cytometry plots of proliferative (EdU positive) cells cultured with additional 200 μM cholesterol incubation for 16 h in different cell densities. *p < 0.05, NS no significance. **f** Free cholesterol assay. ***p < 0.001, NS no significant. **g** Immunofluorescence analysis of cholesterol on single-cell membranes stained by Filipin III. Quantification of the membrane fluorescent intensity is normalized by membrane area (Circled in white line). **p < 0.01, NS no significance. White arrow: Illustrating the cholesterol stained by Filipin III on the cell membrane. White dashed line: Illustrating the area of each cell used for statistical analysis of the cell membrane cholesterol level. **h**, **i** Abundance of cholesteryl esters in cells detected by untargeted lipidomic analysis using high-performance liquid chromatography coupled with mass spectrometry (HPLC-MS/MS). *p < 0.05.

To investigate the molecular regulation of CDC25A during the quiescent state, we used IP-MS to identify molecules interacting with CDC25A. We found that many metabolic pathways were enriched in quiescent state regulated by CDC25A, which is different from the quiescent stem cells with low metabolism and classical function of CDC25A (Fig. 4a, red). Given the importance of lipid metabolism in malignant progression and limited research in quiescent cancer cells, we conducted experiments to explore phenotypic differences in lipid metabolism, including non-esterified free fatty acid, triglyceride, and total cholesterol assays (Supplementary Fig. 3a, b). However, only the total cholesterol level in the *CDC25A*-WT HeLa 100%+2Days group was consistently higher than those in the HeLa 50% group and *CDC25A*-sgRNA HeLa 100%+2Days group (Fig. 4b, d). Similarly, the cholesterol level in the MEF 100%+2Days group was significantly higher than proliferative groups (Supplementary Fig. 3c). The cholesterol levels in the medium are contrary, (Fig. 4c), suggesting cellular cholesterol levels and the capacity of absorbing cholesterol in the quiescent state was elevated compared to the proliferative state. For rescue, we added 200 µM cholesterol in the medium with no extra apoptosis (Supplementary Fig. 3d), the intracellular cholesterol level of the HeLa *CDC25A*-WT 50% group and HeLa *CDC25A*-sgRNA 100%+2Days group prominently increased (Fig. 4d), and the proliferation of the HeLa *CDC25A*-WT 50% group significantly decreased, rather than the *CDC25A*- sgRNA 50% group (Fig. 4e). The higher proliferative rate of CDC25A-sgRNA cells (Fig. 2k, l) requires cholesterol for membrane biogenesis during mitosis [38, 39]. These results suggest that CDC25A might play a role in regulating the homeostasis and transportation of cholesterol in the quiescent state.

Cholesterol contributes substantially to the proliferation, migration, and invasion of cancer [40], existence as free cholesterol(FC) mainly at the plasma membrane impacting the biophysical properties and cholesteryl ester(CE) stored in lipid droplets which can timely converted [40–43]. Therefore, we measured the intracellular levels of FC and CE, respectively. The total FC of the HeLa *CDC25A*-WT 100%+2Days group was significantly higher than that of the HeLa *CDC25A*-WT 50% group, but no obvious changes were observed compared with the HeLa *CDC25A*-sgRNA 100%+2Days group (Fig. 4f). However, after high-density cultivation, FC on the plasma membrane was richer, especially at the cell-cell junction and contact edge (Fig. 4g, white arrows) and obviously decreased after *CDC25A* deficient (Fig. 4g). Furthermore, most of the detected CE in the HeLa *CDC25A*-WT 100%+2Days group were higher than those in the HeLa *CDC25A*-sgRNA 100%+2Days group (Fig. 4i); however, there was no obvious difference between 50% groups (Fig. 4h). Hence, our data suggested that in the quiescent state, CDC25A could upregulate both FC and CE but was more important in the regulation of free cholesterol on the membrane.

### Elevated cholesterol in quiescent cells enhances mechanical resistance and malignant survival

In order to investigate the meaning of enhanced cholesterol in quiescent cells for lung metastasis, we noticed that mechanical force emerged as a significant stressor in high-density cultivation model and metastasis in circulatory system. The tremendous changes of cell shape and resistance of shear stress in circulatory system necessitated the coordination of cell membrane and cytoskeletal changes. Next, we explored the contribution of membrane cholesterol increased by CDC25A to cell membrane and cytoskeletal changes, thereby enhance resistance to mechanical force from crowded environment and circulatory system.

**Fig. 5:**
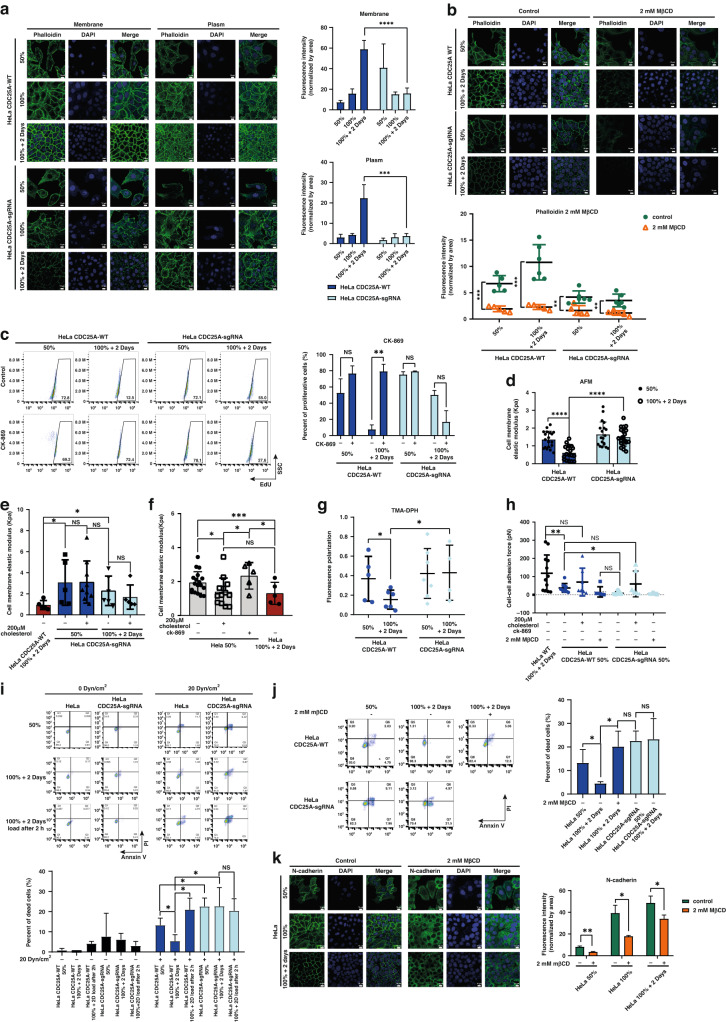
Elevated cholesterol in quiescent cells enhances mechanical resistance and malignant survival. Cytoskeleton on membrane and in plasm were stained by phalloidin in **a**, and with 2 mM MβCD treatment for 16 h in **b**, Quantification of the fluorescent intensity of phalloidin was normalized by area. **p < 0.01, ***p < 0.001, ****p < 0.0001. **c** Flow cytometry plots of proliferative (EdU positive) cells cultured with additional 200 μM CK-869 for 16 h, in different cell densities. **p < 0.01, NS no significance. Rigidity detection of cell membrane expressed as the elastic modulus by AFM in **d**, cells cultured with additional 200 μM cholesterol in **e**, mix of 200 μM cholesterol and CK-869 in **f**, *p < 0.05, ***p < 0.001, ****p < 0.0001. **g** Cell membrane fluidity assay with TMA-DPH. *p < 0.05. **h** Cell-cell adhesion force detection by FluidFM cultured with additional 200 μM cholesterol or CK-869. *p < 0.05, **p < 0.01. **i** Flow cytometry plots of apoptosis assay after shear stress treatment (0 and 20 Dyne/cm^2^) of suspended HeLa *CDC25A*-WT cells and HeLa *CDC25A*-sgRNA cells in different cell densities. 100%+2Days load after 2 h: shear stress was applied on the suspended 100%+2Days cells after 2 h from digestion. *p < 0.05, NS: no significance. **j** Assay like **i**, cultured with additional 2 mM MβCD incubation for 16 h. *p < 0.05, NS no significance. **k** Immunofluorescence analysis of N-cadherin on membrane, cultured with extra 2 mM MβCD for 16 h. Quantification of the fluorescent intensity was normalized by membrane area. *p < 0.05, **p < 0.01.

The cytoskeleton was significantly stronger in the HeLa *CDC25A*-WT 100%+2Days group compared to the proliferative state and *CDC25A*-sgRNA cells, both at the membrane and in the cytoplasm (Fig. 5a). Importantly, the cytoskeleton was significantly decreased after treatment with MβCD, a cholesterol-depleting reagent (Supplementary Fig. 4a and Fig. 5b). Furthermore, the proliferative capacity was significantly increased by treatment with CK-869, a cytoskeleton inhibitor, in the HeLa *CDC25A*-WT 100%+2Days group (Fig. 5c). Together, these data demonstrate that cholesterol upregulated by CDC25A reinforces the cytoskeleton in the quiescent state.

To explore the functional link between upregulated cholesterol and membrane properties, we first examined membrane rigidity using atomic force microscopy (AFM) [44]. The membrane rigidity of the HeLa *CDC25A*-WT 100%+2Days group was significantly lower than that of the *CDC25A*-WT 50% and *CDC25A*-sgRNA 100%+2Days groups (Fig. 5d). The addition of extra cholesterol in the medium (Supplementary Fig. 4a), significantly decreased membrane rigidity in the HeLa *CDC25A*-WT 50% group (Fig. 5f). The reduction in membrane rigidity due to the additional cholesterol in quiescent state was nullified in the absence of *CDC25A* (Fig. 5e), and was also largely reversed by the cytoskeleton inhibitor CK-869 (Fig. 5f). Moreover, we detected the expression of MYH9, encoding the actomyosin cortex under the cell membrane as a determinant of the membrane rigidity [44, 45], was reduced as the cell density increased (Supplementary Fig. 4b, c), and conversely, MYH9 expression became stronger after treatment with MβCD (Supplementary Fig. 4c, d). Similarly, excess cholesterol inhibited the expression of MYH9 (Supplementary Fig. 4e), indicating membrane rigidity reduction in quiescent state due to high cholesterol levels. Besides, cholesterol emerged as a key element of membrane fluidity. Thus we examined the fluidity of membrane by 1-[4 (trimethylamino) phenyl]-6-phenyl-1,3,5-hexatriene (TMA-DPH) [46], which was significantly higher in quiescent state(Fig. 5g). Altogether, the up-regulated cholesterol by CDC25A decrease the rigidity and increase the fluidity of membrane in quiescent state.

Next, we explored the role of cholesterol in the shear stress resistance of the circulatory system. Firstly, previous studies have observed that circulating tumor cells are also multicellular clusters and result in greater capacity of survival in the patient’s circulatory system and chemotherapy evasion [17, 47]. Thus, we measured the adhesion force between living single cells using fluidic force microscopy (FluidFM) and found that the adhesion force in quiescent state was significantly stronger than in the proliferative state, and after cholesterol rescue, the intercellular adhesion force of proliferative state was increased to levels nearly equivalent to those in the quiescent state (Fig. 5h). Secondly, to directly confirm the shear stress resistance, we applied fluid shear stress into floating cells mimicking the circulatory system. After floating, the cells in the 100%+2Days group remained quiescence for about extra 6-8 h without obvious proliferation (Supplementary Fig. 4f, g). Under fluid shear stress, the percentage of dead and apoptotic cells in the *CDC25A*-WT 100%+2Days group was obviously lower than that in the *CDC25A*-WT 50% group (Fig. 5i). The percentage of dead and apoptotic cells was tremendously increased in *CDC25A*-sgRNA HeLa cell lines (Fig. 5i). Following MβCD treatment, the percentage of dead and apoptotic cells in the *CDC25A*-WT 100%+2Days group significantly increased (Fig. 5j). These findings confirm that CDC25A-induced cholesterol elevation enhances resistance to shear stress, supporting the survivability and metastatic potential of the malignant process.

In addition, chemotherapy resistance may be influenced by the changed membrane properties. We treated the cell with paclitaxel, a microtubule-stabilizing agent inducing apoptosis [48], and HeLa *CDC25A*-WT 100%+2Days group exhibited stronger chemoresistance compared to HeLa *CDC25A*-sgRNA cells (Supplementary Fig. 4h). To better understand the relationship between the up-regulated cholesterol and chemotherapy resistance, we treated the cells with a low concentration of paclitaxel for 2 months to enhance the chemoresistance (Supplementary Fig. 4i). The chemoresistance was prominently decreased after incubation with MβCD (Supplementary Fig. 4i). Thus, chemotherapy resistance is also promoted by the accumulation of cholesterol due to increased CDC25A in quiescent cells. Along this line, we observed the expression of the metastatic marker N-cadherin in quiescent cells was higher than that in proliferative cells, and decreased after cholesterol depletion (Fig. 5k). In summary, the upregulation of cholesterol by CDC25A in quiescent state may reinforce the cytoskeleton and adhesion force of cells, while softening the membrane to resist the mechanical force and chemotherapy, thereby promoting the metastasis.

### Correlation Annexin A1 with CDC25A to maintain cholesterol homeostasis by regulating the endosome pathway in quiescent state

**Fig. 6:**
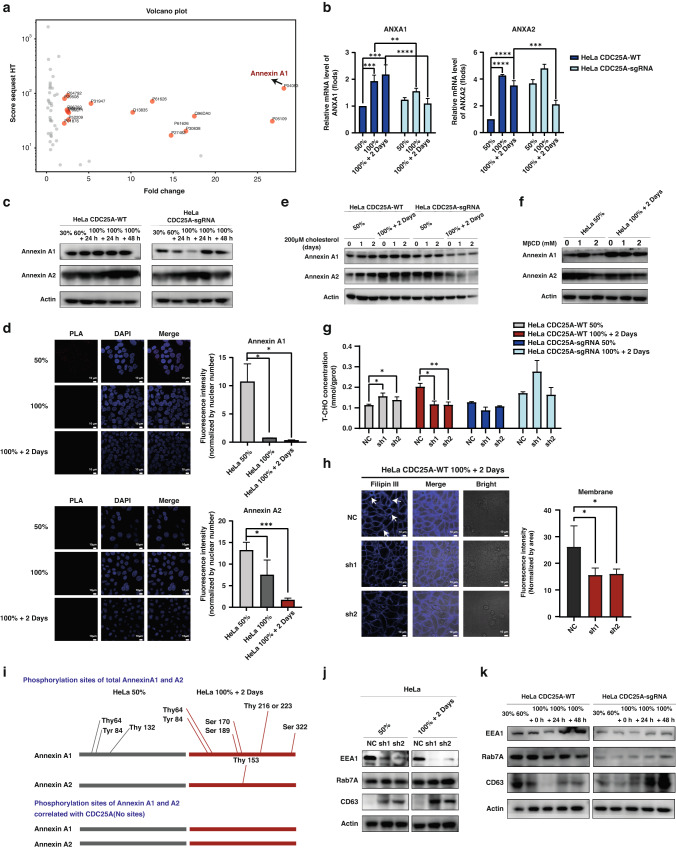
Annexin A1 and A2 are regulated by CDC25A to maintain cholesterol homeostasis in quiescent cell. **a** Volcano plot analysis of the total proteins correlated with CDC25A detected using IP-MS. Compared with HeLa 50%, the proteins in HeLa 100%+2Days group with fold change >2 and score sequest HT > 10 was marked in orange. **b**, **c** RT-qPCR analysis and Western Blot analysis of the expression of Annexin A1 and Annexin A2. **p < 0.01, ***p < 0.001, ****p < 0.0001. **d** The correlations between CDC25A and Annexin A1 and A2 were detected by proximity ligation assay (PLA), red dots in HeLa cells with different densities. Quantification of the fluorescent intensity was normalized by nuclear number. *p < 0.05, ***p < 0.001. **e**, **f** Western Blot analysis of the expression of Annexin A1 and Annexin A2 cultured with extra 200 μM cholesterol and MβCD incubation for 16 h. **g** Total cholesterol assay in cells with Annexin A1 konckdown. *p < 0.05, **p < 0.01. **h** Immunofluorescence analysis of cholesterol on single cell membrane stained by Filipin III in HeLa with Annexin A1 konckdown. Quantification of the membrane fluorescent intensity was normalized by membrane area like Fig. 5g. White arrow: Illustrating the positions with higher levels of stained cholesterol on the cell membrane. *p < 0.05. **i** The phosphorylation sites of Annexin A1 and A2 before and after interacting with CDC25A were identified by IP-MS. **j**, **k** Western Blot analysis of the proteins in endosome pathways.

Next, we sought to elucidate the molecular mechanisms underlying the interaction between CDC25A and cholesterol on the membrane to enhance the mechanical resistance. We identified differentially abundant proteins correlated with CDC25A through IP-MS by comparing the HeLa 100%+2Days group with the HeLa 50% group. Consequently, Annexin A1 was identified for its prominent expression in the HeLa 100%+2Days group (Fig. 6a). KEGG enrichment analysis of the highly expressed proteins in the HeLa 100%+2Days group correlated with CDC25A and were mainly enriched in metabolic pathways(green), cell cycle blocking(orange) and mechanical force regulation(blue) (Supplementary Fig. 5a), indicating the regulation and function of cholesterol in quiescent state.

Additionally, another member of the Annexins family-Annexin A2 was also detected through IP-MS, both the gene transcriptional (Fig. 6b) and protein level (Fig. 6c) of Annexin A1 and A2 were significantly increased in HeLa *CDC25A*-WT cells as cell density increased, and conversely, decreased in HeLa *CDC25A*-sgRNA groups, suggesting that CDC25A induced the expression of Annexin A1 and A2. Notably, we validated the correlation between Annexin A1 and CDC25A (Supplementary Fig. 5b), and despite the enhanced expression, the correlation declined in the HeLa 100%+2Days group (Fig. 6d). Further, the expression of Annexins was induced in the presence of CDC25A with extra cholesterol treatment (Fig. 6e) and decreased with cholesterol reduction by MβCD treatments (Fig. 6f). Moreover, in the *ANXA1* knockdown cell line (Supplementary Fig. 5c), total cholesterol was declined in the HeLa *CDC25A*-WT 100%+2Days group (Fig. 6g), as did the membrane cholesterol (Fig. 6h), however, total cholesterol did not exhibit obvious changes when *CDC25A* was also deficient. These results indicate that Annexin A1, induced by CDC25A but not in combination, plays a vital role in upregulating cholesterol metabolism and maintaining homeostasis.

We next explored the regulation of the Annexins and cholesterol metabolism. As previously reported, Annexins are associated with a membrane structure in endocytic pathways, which is a cholesterol-sensitive process [49]. Cholesterol promotes the phosphorylation of Annexins association with endosome membranes and can be transferred through directly binding. Intriguingly, CDC25A is a dual-specificity phosphatase, and the endocytosis pathway has been enriched by KEGG (Supplementary Fig. 5a). Thus, we hypothesized that the reduced interaction between CDC25A and Annexins (Fig. 6d) in the HeLa 100%+2Days group enhanced the phosphorylation of Annexin A1 and A2 to facilitate transferring membrane cholesterol through endocytic pathways. To test this hypothesis, we performed a phosphorylation site assay using IP-MS. No phosphorylation sites of Annexin A1 and A2 were detected after immunoprecipitation with CDC25A, although they were present in the total Annexin A1 and A2. Consistently, the phosphorylation sites increased in the 100%+2Days group, where the interaction between Annexins and CDC25A was diminished (Fig. 6i). Moreover, the levels of endosome and MVB-related proteins (EEA1, Rab7A, and CD73) were altered due to *CDC25A* and *ANXA1* deficiency (Fig. 6j, k). Evidently, membrane cholesterol is regulated by the phosphorylation of Annexins through the endosome pathway dependent on CDC25A.

### The lipid changes in the quiescent state are related to the cell membrane properties

To further elucidate the function of increased cholesterol in quiescent cells, we performed an untargeted metabolomic assay and lipidomic assay using HPLC-MS/MS to assess the global metabolic and lipid landscape.

Compared with proliferative cells, the up-regulated metabolites in quiescent cells were predominantly rich in some energy metabolism (Supplementary Fig. 6a). As anaerobic glycolysis and aerobic mitochondrial respiration were the two main ways of supplying cellular energy, we assessed the changes in the extracellular media acidification rate (ECAR) and oxygen consumption rate (OCR), between the proliferative and quiescent groups. We consistently observed an increase in glycolysis in the HeLa *CDC25A*-WT 100%+2Days group and starvation for 3 days (Supplementary Fig. 6b, c). The capacity for glucose uptake, accumulation of lactic acid in HeLa *CDC25A*-WT 100%+2Days group (Supplementary Fig. 6d, e) and the expression of pAMPK were elevated (Supplementary Fig. 6f). In addition, we performed an OCR assay and found that the HeLa *CDC25A*-WT 100%+2Days group and starvation treatment was lower (Supplementary Fig. 6g, h). Similarly, the activity of the pyruvate dehydrogenase complex (PDH) decreased (Supplementary Fig. 6i, j). These results demonstrate the energy production and survival in quiescent cells could depend on glycolysis rather than oxidative phosphorylation within the crowded and nutrient-starvation environments.

According to the untargeted lipidomic analysis, we observed that the significantly up-regulated lipids in the HeLa *CDC25A*-WT 100%+2Days group were mainly related to the structure and fluidity of the plasma membrane, cell adhesion, substance transport, and signal transduction (Supplementary Fig. 6k). These data demonstrate that the metabolism of quiescent state is characterized by elevated glycolysis but reduced oxidative phosphorylation. This metabolic profile, adaptive to the less active state, significantly differs from the proliferative state. These observations prompt us to investigate the biological significance of lipid changes related to the cell membrane properties in the quiescent state.

### Metabolism regulated by CDC25A in quiescent state is not related to the energy supply

In order to further understand the metabolic regulation of quiescent cells by CDC25A, we also tested the ECAR and OCR of HeLa *CDC25A*-sgRNA cells at the same time. No obvious change in the ECAR of the HeLa *CDC25A*-sgRNA 50% group compared to that of the *CDC25A*-WT 50% group was observed. However, the glycolytic capacity was decreased and OCR was increased in the HeLa *CDC25A*-sgRNA 100%+2Days group compared with *CDC25A*-WT counterpart (Supplementary Fig. 7a, b), in consistent with the stronger proliferation of the HeLa *CDC25A*-sgRNA cell line (Fig. 2j–l). As for energy, the total ATP content was significantly decreased in the HeLa *CDC25A*-sgRNA 50% group and HeLa 100%+2Days groups compared with *CDC25A*-WT counterpart (Supplementary Fig. 7c). These carbohydrate metabolism and energy changes excluded the possibility that the cholesterol regulated by CDC25A was involved in the energy supply in the quiescent state. Further, we integrated untargeted metabolomics and lipidomic analysis of the difference between the *CDC25A*-WT and *CDC25A*-sgRNA groups. Metabolites that were significantly decreased in the CDC25A-sgRNA group were mainly enriched in pathways related to cholesterol, such as nicotinamide metabolism (Supplementary Fig. 7d, red point). And the main increased proportion of changed lipids in the 100%+2Days groups were related to cell membrane function, cell shape, metabolism and so on, compared with the 50% groups by lipidomic assay (Supplementary Fig. 7e, f).

The metabolic landscape, integrated with the cholesterol assay (Fig. 4) and cellular morphology changes (Fig. 1a, b), suggested that the *CDC25A*-induced cholesterol metabolism increased was not related to the energy supply, but to physical properties of membrane changes.

## Discussion

Quiescence is a reversibly and non-proliferatively poised state orchestrated by precisely intrinsic and extrinsic mechanisms [50, 51]. It encompasses various fundamental physiological processes, including cancer stem cell maintenance, the differentiation of nerve cells [33], oocyte maturation [52], and the creation of immune resistance niches [15, 53, 54]. Regrettably, due to the absence of well-defined models and markers for quiescent cells, the current understanding of these cells and their involvement in metastatic cascades remains limited. In this study, we have established a high-density cultivation quiescent model to mimic the crowded conditions experienced by disseminated cancer cells originating from solid tumors. Our findings provide mechanistic insights into quiescence and malignant progression, enlightening us a new perspective and clinical translational target for metastasis and chemotherapy resistance.

Accordingly, the lipidomic analyses suggest that phospholipid and sphingolipid in the quiescent state also participating in adapting to various environments [55–58]. The non-classical function of CDC25A increasing cholesterol also have other signal pathways, not only the Annexins family, and every phosphorylation site of Annexins, related to the interaction with CDC25A, still have exploration potential. As for mechanical force delivery, the cytoskeleton linking to the nucleus through the nucleoskeleton and cytoskeleton (LINC) complex could regulate nuclear function [59], acting as a potential sensor in response to spatial constraints and regulator of the quiescent. Importantly, the scRNA-seq analysis revealed that a significant portion of cancer cells were capable of entering a quiescent state, different from the previous study identified only a small fraction of quiescent cells such as cancer stem cells, disseminated cells and drug tolerant persister cells. These findings suggest that cancer cells possess remarkable plasticity to adapt and withstand survival pressures in crowded environments in quiescence state, and CDC25A is the potential marker of quiescent state. From a clinical perspective, targeting CDC25A-regulated cholesterol may represent a promising therapeutic approach for addressing circulatory tumor cell dissemination. In future, we can integrate spatial transcriptomics to enhance the diagnostic sensitivity and accuracy of pre-metastasis and metastasis-stages.

## Method details

### Western blot

The indicated cells were lysed by RIPA lysis buffer (Beyotime, # P0013B) on ice for 30 min. After centrifugation at 12000 rpm for 15 min at 4 °C, the supernatant was collected and the protein concentration was quantified by BCA assay (Beyotime, #P0011). 30 μg proteins were separated by SDS-PAGE and transferred onto polyvinylidene fluoride. The membranes were incubated with anti-CDK4 antibody (Cell Signaling Technology,#12790), anti-pRb antibody (Cell Signaling Technology, #8516),anti-CDK6 antibody (Cell Signaling Technology, #13331), anti-β-actin antibody (proteintech, #81115-1-RR), anti-CDC25A antibody (proteintech, #55031-1-AP), anti-cas9 antibody (Abclonal, A23005), anti-m-TOR antibody (Cell Signaling Technology, #5536), anti-Phospho-AMPKα (Cell Signaling Technology, #2535),anti-Annexin A1 antibody (Cell Signaling Technology, #32934), Annexin A2 antibody (proteintech, #66035-1-Ig), anti-EEA1 antibody (abcam, #ab109110), anti-Rab7 antibody (Biotechnology, # 55469-1-AP), or anti-MYH9 antibody(abcam,#ab138498).

### Real-time quantitative PCR (RT-qPCR)

Total RNAs were isolated from the cells or mouse tissues using a TRIzol reagent. The cDNA was synthesized from 2 µg of total RNA using the PrimeScript™ II 1st Strand cDNA Synthesis Kit (Takara, #6210A) and subjected to RT-qPCR with TB Green® Premix Ex Taq™ (Tli RNaseH Plus) Kit (Takara, #RR420A). Primary sequence: CDC25A: F-TTCCTCTTTTTACACCCCAGTCA, R-TCGGTTGTCAAGGTTTGTAGTTC; β-actin: F-CATGTACGTTGCTATCCAGGC, R-CTCCTTAATGTCACGCACGAT; Annexin A1: F-CTAAGCGAAACAATGCACAGC, R-CCTCCTCAAGGTGACCTGTAA; Annexin A2: F-GAGCGGGATGCTTTGAACATT, R-TAGGCGAAGGCAATATCCTGT. The relative expression of the target genes was normalized to β-actin mRNA.

### Spheroid formation

HeLa and HeLa CDC25A-sgRNA cell line were transfected with U6-MCS-Ubiquitin-Cherry-IRES-Blasticidin lentivirus, seeded into Corning® 96-well Spheroid Microplates (#4520, 150 cells/well). After 2 weeks incubation with 1% methylcellulose completed DMEM, the images were captured by Cell Voyager CV800(20x Water, YOKOGAWA, Japan).

### Fluidic force microscopy (FluidFM) Assay

The adhesion force between living single cells was detected using fluidic force microscopy (FluidFM, OMNIUM, Quantum Design China). A FluidFM system composed of force-controlled cantilevers with an incorporated microfluidic channel and connected to a digital pressure controller using the EasyScan2 software. A 4 μM micropipette probe with microfluidic channels (Cytosurge AG, Swizterland, #14363) was used, and the nominal spring constant was 0.3 N/m. The cells were seeded in a 12 well plate (Costar, #3513) and co-incubated with the another just digested cell at 37 °C for 15 min. The medium was gently removed, and the non-adherent cells were washed with PBS 2-3 times. The just adherent cells were absorbed by the micropipette probe, and the adherent force between single cells was detected. Some parameter settings were based on previously published studies [60]. All groups were measured at 37 °C within 1.5 h.

### Cell membrane fluidity by TMA-DPH

Membranes could be labeled with TMA-DPH (1-(4-trimethylammoniumphenyl)-6- phenyl-1,3,5-hexatriene p-toluenesulphonate) according to BBcellProbe® TMA-DPH Kit (Bestbio BB-48118). Dilute the TMA-DPH probe 1000 times with diluent to prepare the TMA-DPH staining working solution. Digested and resuspended cells with staining solution and incubated at 37 °C for 15-30 min. Washed cells once with PBS and resuspended cells into black 96-plate (10^5^ cells/100ul/well). The change of fluorescence polarization was detected by microplate reader.

### Cell exposure to shear stress

HeLa *CDC25A*-WT and HeLa *CDC25A*-sgRNA cells were digested by 0.5% trypsin and resuspend with 2 ml completed DMEM, transferred to a new 6-cm Corning plate. The plate was placed in a cone-plate shearing system immediately (unless otherwise specified) and the cells will be subjected to shear stress during the cone spinning. The shear stress was set to 20 dyn/cm^2^ [61].

## Supplementary information

Supplemental material

## Data Availability

We sincerely appreciate for the assistance with Dr. Siying Qin for technical help with the Core Facilities at School of Life Sciences, Peking University and the help at State Key Laboratory of Natural and Biomimetic Drugs, Peking University, Beijing, China; the Dr.Wen Zhou at Analytical Instrumentation Center in Peking University for assistance with IP/MS. All data are available in the main text or the supplementary materials. Single-cell RNA sequencing data are available with accession number HRA008564 (https://ngdc.cncb.ac.cn)

## Code availability

Coding analyses for single-cell RNA sequencing data are available in Scanpy with the URL accession link https://scanpy.readthedocs.io/en/stable/api/index.html
