# Supplementary material for: Quiescent cancer cells induced by high-density cultivation reveals cholesterol-mediated survival and lung metastatic traits
Source: Br J Cancer. 2024 Oct 11;131(10):1591–604. doi: 10.1038/s41416-024-02861-x (PMC11555385; doi:10.1038/s41416-024-02861-x)
Supplement: Supplementary file 1 — Supplemental material [file 41416_2024_2861_MOESM1_ESM.docx]

Supplementary Materials

**MATERIALS AND METHODS**

**Mice**

4/5-weeks-old BALB/c-nu females mice brought from Beijing HFK Bioscience CO.,LTD were completely randomized and used for all experiments as tumor-bearing mice and lung metastatic mice.

**Cell lines**

The human ESCC cell lines Yes 2, human cervical carcinoma cell line HeLa and Mouse Embryonic Fibroblast (MEF) cell lines were used for experiments. MEF cell was isolated from embryos of C57 mice at 13.5 days of pregnancy. Yes 2 was cultured in RPMI-1640 Medium, 10% FBS, 1% Penicillin-Streptomycin. Wide type HeLa and engineered HeLa cell line were cultured in DMEM with High Glucose, 10% FBS, 1% Penicillin-Streptomycin. MEF cell line was cultured in DMEM with High Glucose, 10% FBS, 1% Penicillin-Streptomycin, 1% NEAA(Gibco), 1% Sodium pyruvate (Gibco), 1% GlutaMax(Gibco).

**Lentiviral Transfection**

Annexin A1 shRNA lentivirus (Negative control: U6-MCS-Ubiquitin-Cherry-IRES-Blasticidin) were constructed and purchased from GENECHEM. Culture the cells in a 6-well plate to a confluency of 60% and add 25x transfection reagent P (Genechem, China) and 40 MOI lentivirus for 24 h. Then, change a fresh medium to culture the above cells 2 days, and 3ug/ml blasticidin was added for stable cell line selection.

HeLa CDC25A-sgRNA cell line was generated through CRISPR/Cas9-mediated genome editing. The single guide RNAs (sgRNAs) were designed using the online CRISPR design tool (Red CottonTM, Guangzhou, China, <https://en.rc-crispr.com/>).

gRNA(fW): CGCGTCGCAGCCCGTCGTGAAGG (specificity scores: 98; efficiency scores: 0.69). The gRNA and cas9 lentivirus were constructed and purchased from Ubigene Biosciences Co., Ltd.(Guangzhou, China). The gRNA oligos was annealed and ligated to the YKO-LV005 vector, and the cas9 was annealed and ligated to the YCas-LV001 vector. 72 h after the cas9 lentivirus transfection, 5ug/ml puromycin were added to screen the cells for 2 weeks. Then transfect gRNA lentivirus into cas9 overexpressed cell and 500ug/ml hygromycin B were added to screen the cells for 2 weeks. After antibiotic selection, a certain number of cells were diluted by limited dilution method and inoculated into 96-well plate. Selection of single clones were performed after 2-4 weeks and selected CDC25A deficient clones validated by PCR and Sanger sequencing.

**METHODS DETAILS**

**Isolation and primary cultures of Mouse Embryo Fibroblasts**

After cohabitation of mice, check for vaginal plugs in the morning (before 8 AM). The day of plug detection is considered as 0.5 days, and embryos are collected on day 13.5 of gestation. Under aseptic conditions, euthanize the pregnant mouse, place the mouse with its abdomen side up in a sterile 100mm culture dish. Sequentially, make an incision in the abdominal skin and place the embryos in another sterile 50ml centrifuge tube containing 30ml of PBS. Wash 2-3 times with to remove surface blood. Then, transfer each embryo separately into a new sterile 6-well plate, with one embryo per well. Remove the heads, viscera, and limbs from the embryos, then transfer them to another 6-well plate containing PBS, and repeat the wash until there is no visible red tissue. Transfer the obtained embryo tissues to a sterile 24-well plate, with one embryo per well and mince the tissues (approximately 100 cuts per well). Add 1ml of 0.05% trypsin to each well and gently pipette up and down 10-20 times, then incubate at 37°C for 20-30min and filtrate. Add 1ml of culture medium to each well and use a 1ml pipette tip to dissociate the cells. Transfer the cells to 10cm culture dishes pre-treated with 1% gelatin. Change to fresh culture medium the next morning. Typically, experiments are conducted using cells from P2 to P4.

**Evaluation of cell cycle by EdU**

Before collection, cells were incubated with 10μM (1X) EdU (5-ethynyl-2’-deoxyuridine) for an hour. Then the cells were digested with 0.5% trypsin, fixed with 4% paraformaldehyde and premeabilized with 0.5% Triton X-100 for 15min. Next, incubated the cell with Click Additive Solution according to the manufacture’s instruction for 30min in dark, then removed the Click Additive Solution and washed the cells 2 times before the measurement of BD Accuri™ C6 Plus Flow Cytometer. Data were analyzed using FlowJo v.10 software.

**Evaluation of cell size and Ki-67 signal by flow cytometry**

For the analysis of cell size by forward scatter (FSC), the cells were digested with 0.5% trypsin and fixed with 4% paraformaldehyde with the measurement of BD Accuri™ C6 Plus Flow Cytometer. Data were analyzed using FlowJo v.10 software.

For the analysis of Ki-67 signal, the cells were digested with 0.5% trypsin, fixed with 4% paraformaldehyde and premeabilized with 0.5% Triton X-100 for 15min. Then incubated the cell with anti-Ki-67 antibody (abcam, #ab16667) for an hour at room temperature on a rotator. Next, cells were stained with anti-Rabbit-488nm secondary antibody (Gene-Protein Link, #P03S14M) in dark for 30 min at room temperature on a rotator with the measurement of BD Accuri™ C6 Plus Flow Cytometer. Data were analyzed using FlowJo v.10 software.

**Pyronin Y**

To assay the RNA content of the cells, the cells were digested with 0.5% trypsin, fixed with 4% paraformaldehyde and premeabilized with 0.5% Triton X-100 for 15min. Then incubated the cell with 1U DNase I (Beyotime, # D7076) for 30min at 37°C. After excluding the effects of DNA, cells were stained with 1% Pyronin Y (MCE, # HY-D0971,) for 30min in dark at room temperature. The samples were measured by BD FACS Celesta™ Flow Cytometer and analyzed by FlowJo v.10 software.

**Apoptotic assay**

To assay the percent of dead cells under all kinds of treatment, the cells were digested with 0.5% trypsin and incubated with binding buffer containing Annexin V-FITC/PI (or Annexin V-APC/PI) for 20min in dark at room temperature, according to manufacturer’s instructions of Apoptotic kit (Multi science, #AP107-100, #AP101C-100). The samples were measured by BD Accuri™ C6 Plus Flow Cytometer and analyzed by FlowJo v.10 software.

**Cell synchronization by double thymidine block**

Cell synchronization using double thymidine block has been widely used to study the mechanisms of cell cycle progression. At 40% confluence, cells were treated with 2mM thymidine (MCE, HY-N1150) for 18h (first block) and then switched to normal DMEM for 10 h. After 10h of release, the normal medium was again replaced with 2mM thymidine for 18h (second block), and cell synchronization was completed. Finally, the cells were released with normal DMEM from 0 to 90h; cells at each time point were collected.

**Cell proliferation/viability assay and IC50 values by MTS**

The resistance of lethality of paclitaxel on HeLa CDC25A-WT and HeLa CDC25A-sgRNA cells were evaluated using MTS assay. In brief, 4 x 10^4^ indicated cells in 100 ul of DMEM medium were cultured in each well of 96-well plates. 24h later, cells were treated with different doses of paclitaxel (0-20uM) for 72h. Then, the medium was discarded, and cells were incubated with 10% MTS solution (Cat# G3582; Promega) for 1 h and read by microplate reader at 490 nm. The number of viable cells was positively correlated with formazan. The IC50 values calculation were based on MTS assay.

**Transwell migration assays**

Migration of HeLa CDC25A-WT and HeLa CDC25A-sgRNA cells *in vitro* were assayed by 6.5 mm Transwell chamber systems (Costar, #3422) with polycarbonate membrane inserts containing 8um pore size. The indicated cells (1x 10^5^ cells) were seeded in the upper chamber with 200 ul FBS-free DMEM medium, and the lower chamber was added with 1 ml of DMEM medium with 20% FBS. After 24h, the upper chambers were fixed in methanol for 15min and stained with 1% crystal violet solution for 15min, and the non-migratory cells were removed with cotton swabs. The migratory cells were then photographed under a microscope (Leica DM2500 LED, Leica).

**Colony formation** **assay**

For colony formation assay, 2000 HeLa CDC25A-WT and HeLa CDC25A-sgRNA cells were seeded in 6cm plates for 2–3 weeks, and the colonies were fixed with methanol then stained with 1% crystal violet. The images were captured by UVITEC Cambridge and Essential V6 software.

**Tumor** **models**

Subcutaneous Xenograft Mouse Model. A total of 1×10^6^ of HeLa CDC25A-WT and HeLa CDC25A-sgRNA cells were suspended in 100μl of 30% Matrixgel (Coring, #354248) and subcutaneously injected into the bilateral flanks of BALB/c-nu mice.

Mouse Lung Metastasis Models. A total of 7×10^6^ of HeLa CDC25A-WT and HeLa CDC25A-sgRNA cells were suspended in 150μl of PBS and injected into the tail veins of BALB/c-nu mice. 2.5 months after injection of the cell suspensions, these mice were killed and lung metastasis formation was observed under direct viewing.

**Immunohistochemistry (IHC) analysis**

IHC analysis was carried out to determine altered protein expression in indicated paraffin-embedded mouse bearing tumor and lung metastatic tissue and followed by anti-Ki-67 antibody (abcam, #ab16667) overnight at 4°C. The images were captured by Lecia DM2500 LED and LAS V4.9 software.

**Immunoprecipitation (IP)**

The indicated cells were lysed by RIPA lysis buffer (Beyotime, # P0013B) on ice for 30min. After centrifugation at 12000 rpm for 15min at 4°C, the supernatant was subjected to a new tube, and incubate with protein A/G magnetic beads (MCE, #HY-K0202) for 60min at 4°C. The supernatant incubated with Annexin A1 antibody (proteintech, #21990-1-AP) or Annexin A2 antibody (proteintech, #60051-1-Ig) and protein A/G magnetic beads and rotated overnight at 4°C. The immunoprecipitates were washed seven times with wash buffer (50 mM Tris-HCl [pH 7.4], 0.05% NP-40, 1 mM MgCl_2_, 150 mM NaCl) and added another 50ul RIPA for western blotting.

**scRNA-seq 10X Genomics and Analysis**

**Single-cell RNA-seq Data Preprocessing**

Single-cell sequencing data of HeLa 50% and HeLa 100%+2Days were aligned with the GRCh38 human reference genome and quantified using Cell Ranger (version 3.0, 10x Genomics Inc). The quality of cells was assessed based on two parameters: cells with (1) detected genes fewer than 8000 and larger than 200; (2) mitochondrial unique molecular identifier (UMI) count percentage fewer than 15% were retained. The genes can be detected in more than 3 cells were retained. We next normalized the count data, using the *scanpy.pp.normalize_total* function with parameter “*target_sum=1e4*”.

The highly variable genes (HVGs) were selected using the *scanpy.pp.highly_variable_genes* function with the parameter setting “*min_mean=0.0125, max_mean=4, min_disp=0.2*”. Next, principal component analysis (PCA) was performed using the *scanpy.tl.pca* function and 31 components were used for downstream analyses. Mostly unwanted sources of variation was regressed out with the parameters of *sc.pp.regress_out* function.

**Unsupervised Clustering**

We performed unsupervised clustering to HeLa 50% and HeLa 100%+2Days cell population using the *scanpy.tl.leiden* function with parameter setting “resolution=0.6”. The specific marker genes of clusters were identified by *scanpy.tl.rank_genes_groups* function with the parameter of “*method=wilcoxon*”. The combining plots of highly differential genes were performed by *scanpy.pl.heatmap* and *scanpy.pl.dotplot*.

**Pseudotime Analysis**

The spliced and unspliced UMIs of scRNA-seq data used for pseudotime was recounted by velocyto. The diffusion map was built by the *scanpy.tl.diffmap* function and the pseudotime trajectory inference by *scanpy.tl.dpt* function.

**T-CHO, NEFA and TG assay**

T-CHO (Total cholesterol assay kit, #A111-1-1), NEFA (Nonesterified Free fatty acids assay kit, #A042-2-1) and TG (Triglyceride assay kit, #A110-1-1) assay kits were purchased from Nanjing Jiancheng Bioengineering Institute (China, Nanjing). The indicated cells were lysed by RIPA Lysis Buffer (Beyotime, # P0013B) on ice for 30min and centrifugated at 12000 rpm for 15min at 4°C, whose supernatant was subjected to assay by microplate reader according to manufacturer’s instructions, respectively. The assays were normalized by total concentration of cell protein.

**Free Cholestenone (FC) Content Assay**

Free Cholestenone (FC) Content Assay Kit was purchased from Solarbio Life Science (China). The indicated cells were broken by ice bath ultrasound (power 300W, ultrasound for 2 seconds, interval of 3 seconds, total time of 3min) with extraction solution- isopropanol. Then centrifugated at 8000g for 10min at 4 ℃, whose supernatant was subjected to assay with working buffer by microplate reader (500nm) according to manufacturer’s instructions.

**Pyruvate Dehydrogenase (PDH) Activity Assay**

Pyruvate Dehydrogenase (PDH) Activity Assay Kit was purchased from Solarbio Life Science (China). The indicated cells were broken by ice bath ultrasound (power 200 W, ultrasound 3 s, interval 7 s, total time 5min) with extraction solution according to manufacturer’s instructions. Then centrifugated at 11000g for 10min at 4 ℃, whose supernatant was subjected to assay with working buffer by microplate reader(605nm) according to manufacturer’s instructions.

**Identification of protein by MS analysis**

As for the specific bands of silver staining, proteins obtained by IP as described before were separated by SDS-PAGE and stained with silver staining kit as manufacturer’s instructions (Beyotime, # P0017S). The proteins of specific bands were identified by the Orbitrap Elite Mass Spectrometer (Thermo). As for total proteins obtained by IP, the identification of proteins was measured by Orbitrap Fusion LUMOS Tribrid Mass Spectrometer (Thermo).

**Untargeted metabolomics and untargeted lipidomic detection**

Aqueous metabolite detection (untargeted metabolomics) and lipid phase metabolite detection (untargeted lipidomic) were measured by Ultimate 3000 ultra-high performance liquid chromatography. The mass spectrometry data-dependent acquisition was finished by Q-Exactive HF combined quadrupole mass spectrometer. The data was analyzed by the website of MetaboAnalyst 5.0.

**Seahorse XFe96 Assay**

The Oxygen consumption rate (OCR) and extracellular acidification rate (ECAR) of different group were measured following the manufacturer’s instructions of Seahorse XF Cell Mito Stress Test Kit and Glycolysis Stress Test Kit (Agilent). In brief, 8*10^4^cells were seeded in XF96 plate 3 days advanced for the group of 100%+2Days, 1 days advanced for the group of 50%. 6*10^4^cells were seeded in XF96 plate 5 days advanced for the group of starvation for 4 days and so on. For the group of different density, the OCR and ECAR under basal conditions was response to 4μM oligomycin, 1.5μM trifluoromethoxy carbonylcyanide phenylhydrazone (FCCP, Sigma) and 6μM rotenone and antimycin A during measurement of Seahorse XF Pro (Agilent). For the group of starvation, the OCR and ECAR was response to 3μM oligomycin, 1.2μM FCCP and other conditions remained unchanged. The results were normalized by cell number counted by High Content Assay stained with hochest 33342.

**Glucose Uptake (2-NBD glucose)** **Assay**

Remove the completed medium from cells and replace it with serum free culture medium for 6 h. Then incubate cells with 200μM 2-NBDG (Invitrogen™, #N13195) in PBS buffer for 20min at 37°C, digested with 0.5% trypsin analyzed by flow cytometry.

**Lactic Acid Assay**

The indicated cells were lysed by RIPA Lysis Buffer (Beyotime, # P0013B) on ice for 30min and centrifugated at 12000 rpm for 15min at 4°C, whose supernatant was subjected to assay with kit (biosharp, #BL868A). Preheat the microplate reader for 30min and set the wavelength to 450nm. Working solution was mixed with reagents 1, 2, 3, and 4 in a ratio of 20:10:130:10 according to manufacturer’s instructions. Added 170 μl of ATP assay working solution to each sample (20ul) and incubated in 37°C in dark for 30min, then detected.

**Enhanced ATP Assay**

The indicated cells were lysed by RIPA Lysis Buffer (Beyotime, # P0013B) on ice for 30min and centrifugated at 12000 rpm for 15min at 4°C, whose supernatant was subjected to assay with Enhanced ATP Assay Kit (Beyotime, # S0027). Dilute ATP assay reagent with ATP assay buffer at a 1:4 ratio to create working solution. Add 100 μl of ATP assay working solution to each assay well and incubate at room temperature for 3-5min to exclude endogenous ATP and background signal. Add 20 μl of the sample to each assay well, mix quickly and measure.

**Immunofluorescence (IF) staining**

The indicated cells were plated on 15 mm diameter glass-bottom plates (NEST). For IF staining of CDC25A, phalloidin, Annexin A1, E-cadherin and N-cadherin, the cells were fixed with 4% paraformaldehyde and premeabilized with 0.5% Triton X-100 for 15min, then blocked with goat serum. For IF staining of Filipin III, the cells were just fixed with 4% paraformaldehyde. The cells were incubated with anti-CDC25A antibody (proteintech, #55031-1-AP; Santa Cruz Biotechnology, #sc-7389(PLA)), anti-Annexin A1 antibody (proteintech, #21990-1-AP), Anti-E-cadherin antibody (proteintech, #60335-1-Ig), anti-N-cadherin antibody (proteintech, #22018-1-AP) or anti-MYH9 antibody (abcam,#ab138498) in 4°C overnight；phalloidin (Gene-Protein Link, P11Q04, 1:200) in room temperature for 30min. Then incubated fluorescent secondary antibody in room temperature for 60min and DAPI for 10min in dark.

**Filipin III staining**

Cholesterol of cell membrane is stained by Filipin III. Filipin III was dissolved in ethanol to reach the final concentration of 5mg/ml. Cells were fixed with 4% paraformaldehyde (PFA) and stained with 500 μg/ml filipin III for 30min at room temperature. Images were collected using a ZEISS 780 confocal microscope and analyzed using a ZEISS zen blue software.

**Proximity ligation assay (PLA)**

The indicated cells were plated on 15 mm diameter glass-bottom plates (NEST) and fixed with 4% paraformaldehyde and premeabilized with 0.5% Triton X-100 for 15min, then blocked in Duolink blocking buffer. The cells were incubated with anti-CDC25A antibody (Santa Cruz Biotechnology, #sc-7389) and anti-Annexin A1 antibody (proteintech, #21990-1-AP), anti-Annexin A2 antibody (proteintech, #66035-1-Ig) in 4°C overnight. Then the cells were subjected to in situ PLA according to Duolink® PLA Fluorescence Protocol (MilliporeSigma). In brief, after washing primary antibody, PLUS and MINUS probes were applied, then ligation, amplification, and detection with IF Zeiss LSM780.

**Atomic Force Microscope (AFM) Assay**

AFM force spectroscopy experiments were performed with AFM Bruker BioScope Resolve (Billerica, MA, USA). Force mappings were obtained using a nitride tip on nitride lever (MLCT-SPH-1UM(D), Bruker) with elasticity coefficient of 0.029 N/m. All force curves were analyzed in Nanoscope Analysis software (Bruker) to extrapolate Young’s modulus. All samples were measured at room temperature within 1 h.

**QUANTIFICATION AND STATISTICAL ANALYSIS**

The statistical analyses used in the study included the f-test for equality of variances, Wilcoxon test and one-sided t-test, as described in the Figure legends. Correlations among the groups were estimated using Pearson’s correlation. We obtained the enriched pathways by KEGG enrichment analysis with the “KEGG_2019_Human” reference gene sets and GO_BP enrichment analysis on <https://david.ncifcrf.gov/>.

**
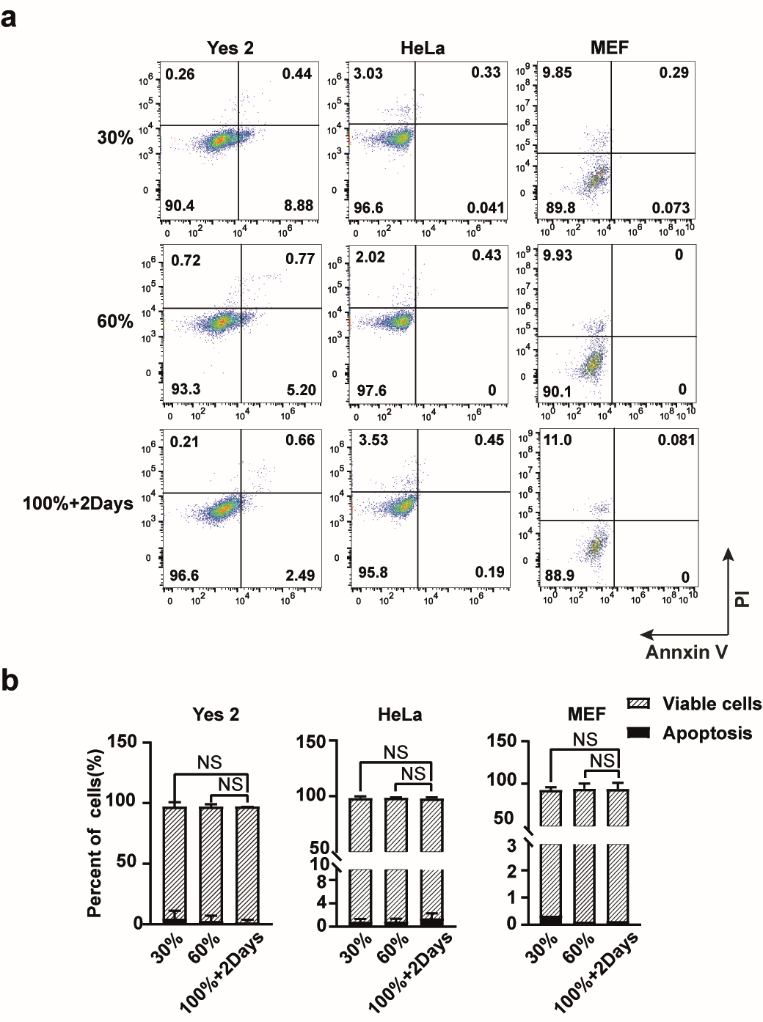
**

Supplementary Fig.1: Apoptotic assessment and identification of different densities.

**a**, Flow cytometry plots of apoptosis assay in different cell densities.

**b**, Quantification of the percent of apoptosic and viable cells. NS: no significance.


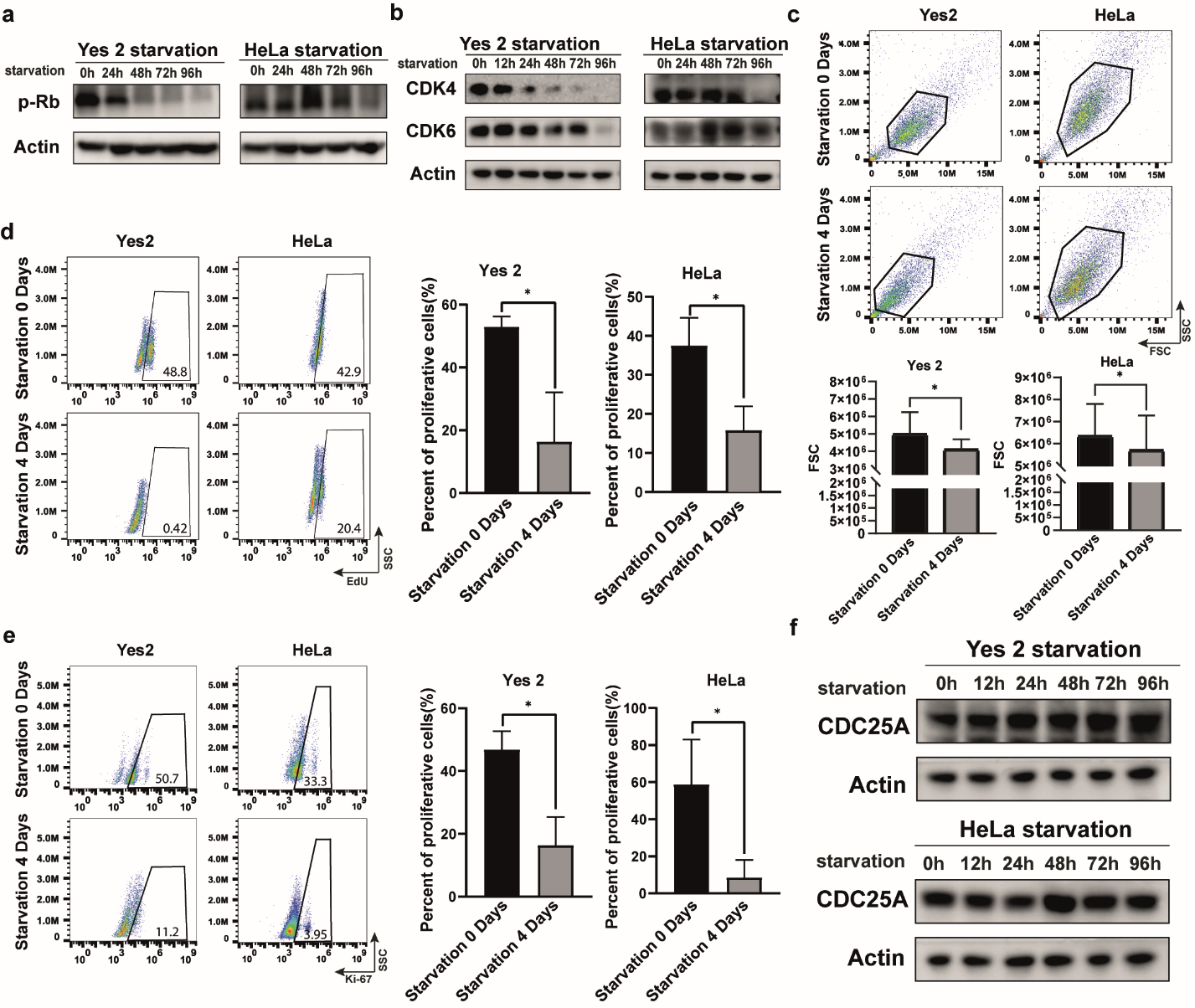


Supplementary Fig.2: Establishment of quiescent model by serum starvation.

**a-b**, Western Blot analysis of the expression of cell cycle related protein after serum starvation for 0 h-96 h. **c**, Flow cytometry plots of cell size after starvation treatment. *p < 0.05. **d-e**, Flow cytometry plots of proliferative EdU positive cells in **d**, Ki-67 positive cells in **e**. *p < 0.05. **f**, Western Blot analysis of CDC25A expression along with the starvation.

**
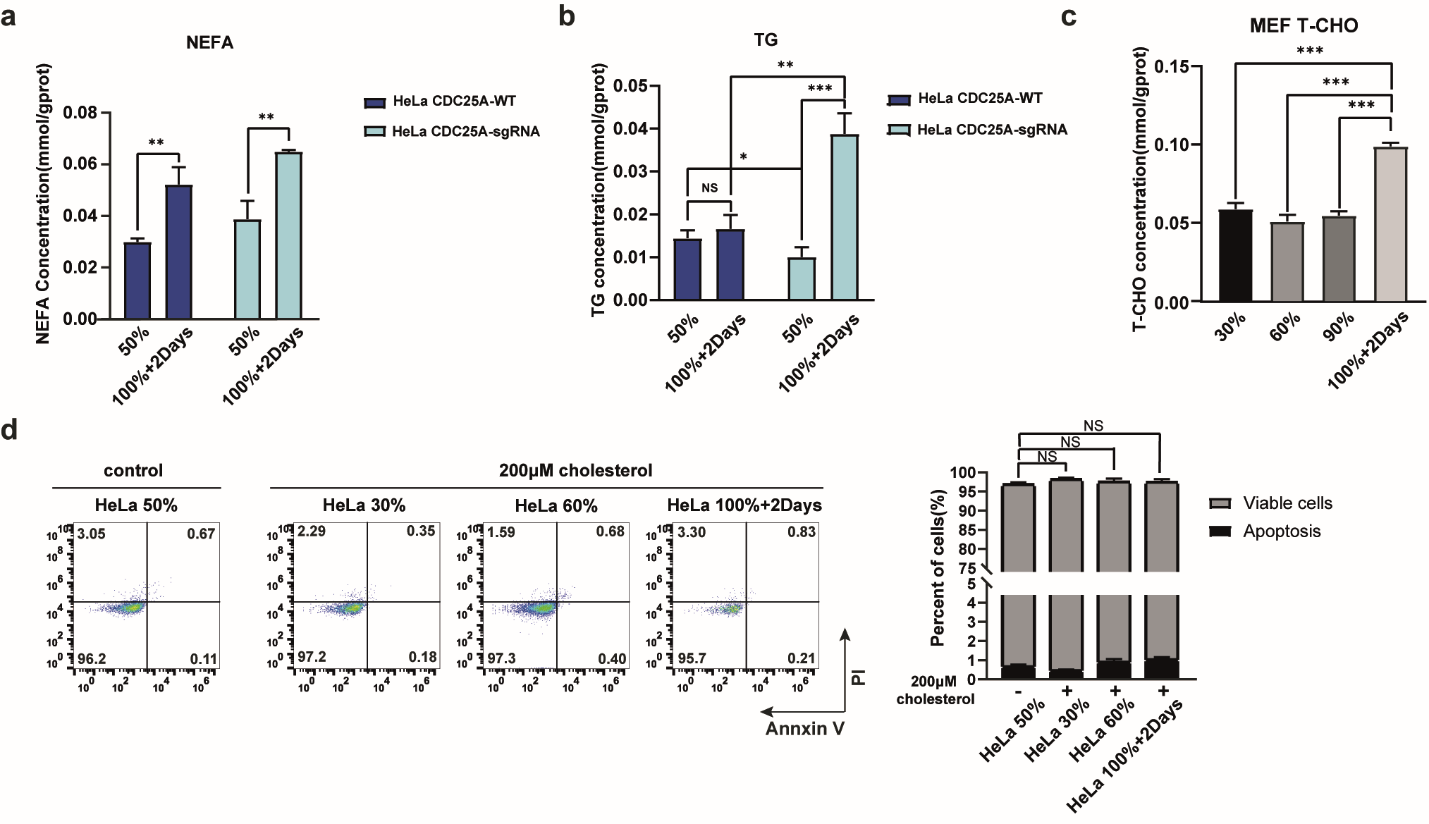
**

Supplementary Fig.3: The lipid metabolite assay and cell survival after cholesterol treatment.

**a-b**, Nonesterified fatty acid (NEFA) assay in **a**, Triglyceride (TG) assay in **b** of HeLa *CDC25A*-WT cell line and HeLa *CDC25A*-sgRNA cell line in different cell densities. *p < 0.05, **p < 0.01. **c**, Total cholesterol assay in different MEF cell density. ***p < 0.001. **d**, Flow cytometry plots of Apoptosis assay after 200μM cholesterol treatment. NS: no significance.

**
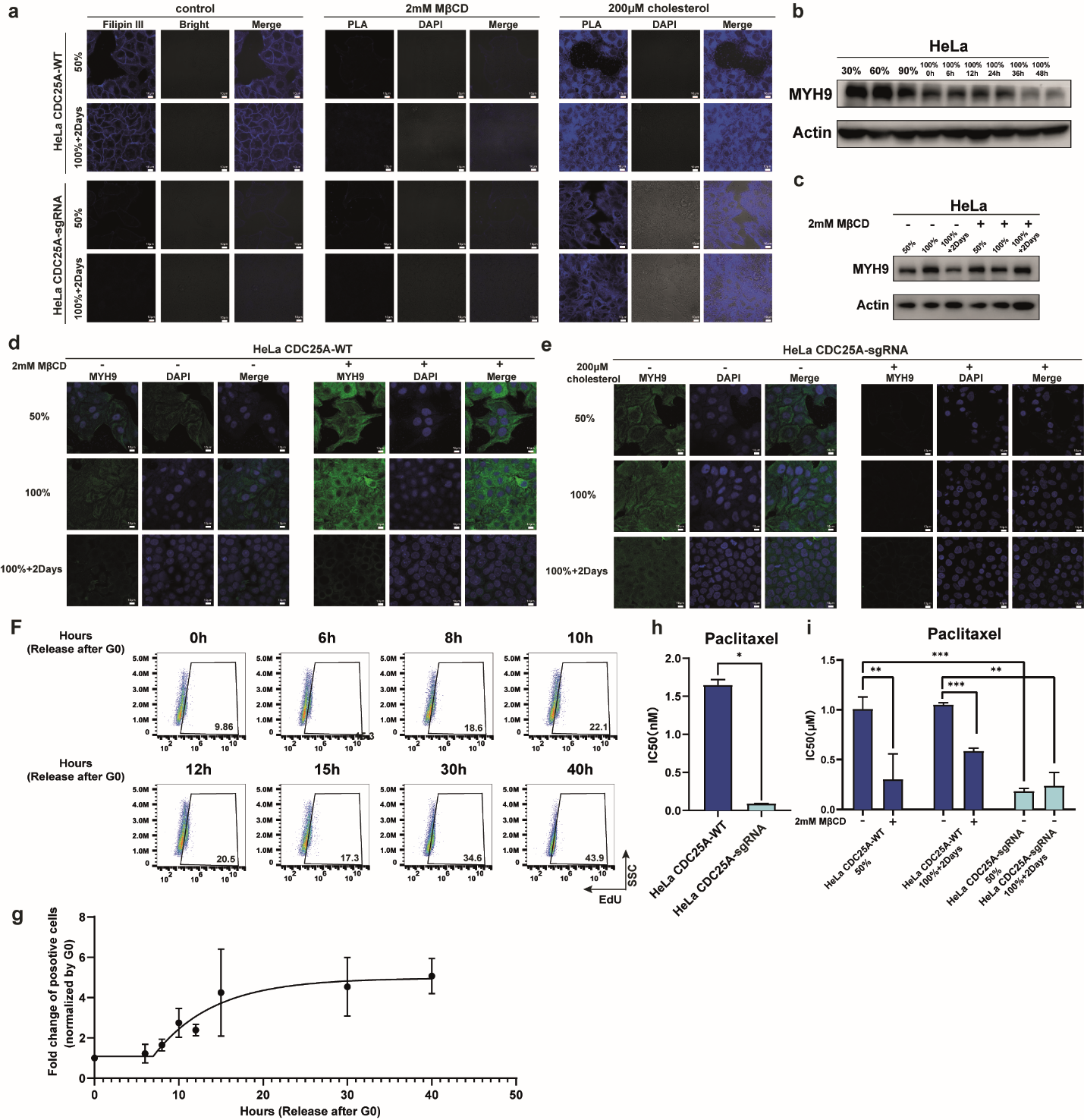
**

Supplementary Fig.4: Cholesterol on membrane and cell cortex analysis under different treatment.

**a**, Immunofluorescence analysis of cholesterol on membrane, cultured with extra 2mM MβCD or 200μM cholesterol incubation for 16 h. **b**, Western Blot analysis of the expression of MYH9 along with the increased cell density. **c**, Western Blot analysis of the expression of MYH9 with 2mM MβCD incubation for 16 h. **d-e**, Immunofluorescence analysis of MYH9 with **d**, 2mM MβCD incubation HeLa *CDC25A*-WT cell line or **e**, 200μM cholesterol HeLa *CDC25A*-sgRNA for 16 h in different density. **f**, Flow cytometry plots of proliferative EdU positive cells of HeLa cells whose density is equal to 100%+2Days. **g**, Fitting curve of the increased proliferative EdU positive along with the time released from quiescent state. **h**, IC50 values(nM) of paclitaxel effects for cells. **i**, IC50 values(μM) of paclitaxel effects for cells with paclitaxel acquired resistance in a certain extent.

**
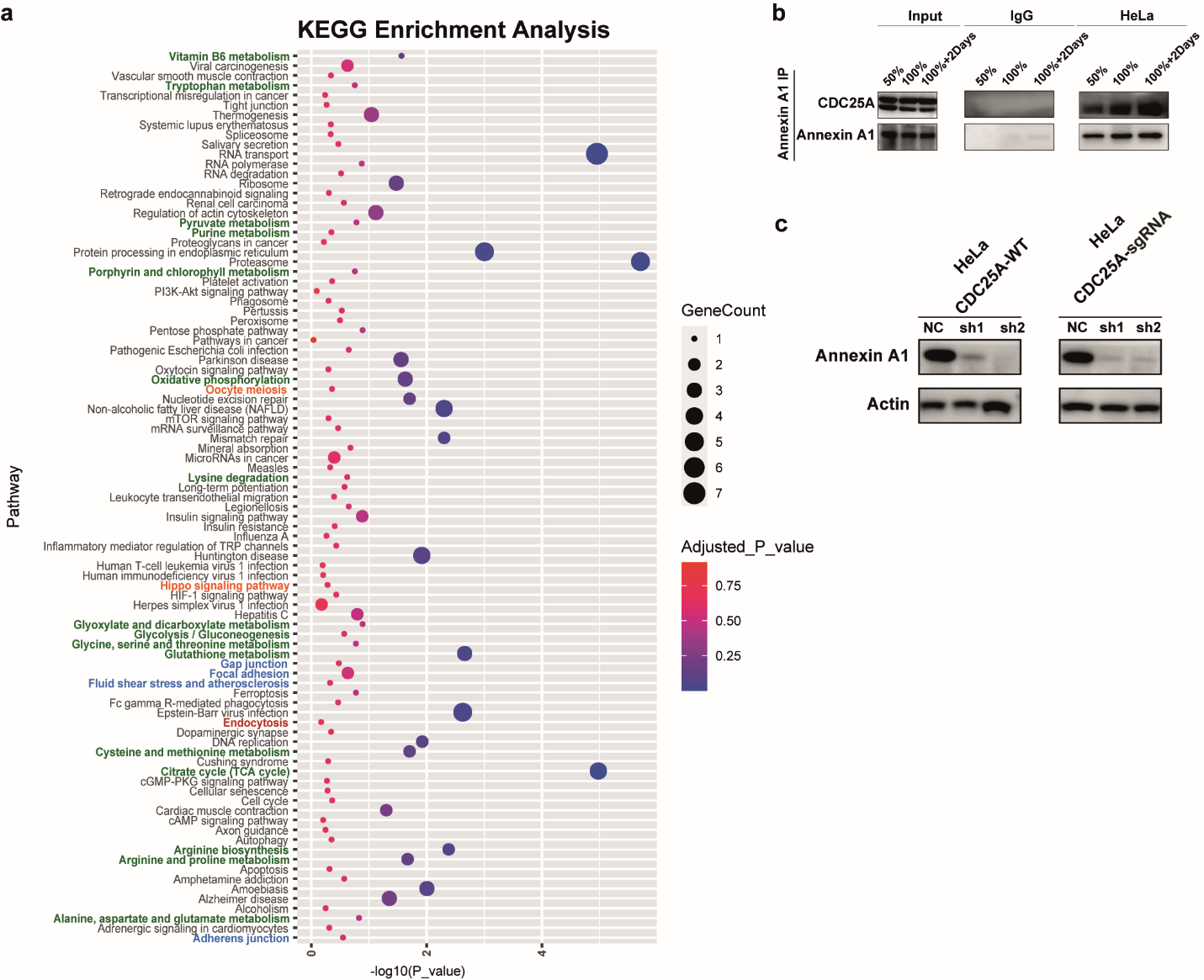
**

Supplementary Fig.5: Verification of the correlation between Annexin A1 and CDC25A.

**a,** KEGG enrichment analysis of the total protein correlated with CDC25A detection using IP-MS. The pathways are related to metabolism(green), mechanical force(blue), cell cycle block (orange) and so on(black). **b,** Western Blot analysis of the correlation of Annexin A1 and CDC25A. **c,** Western Blot analysis of the contraction of Annexin A1-Knockdown cell line.

**
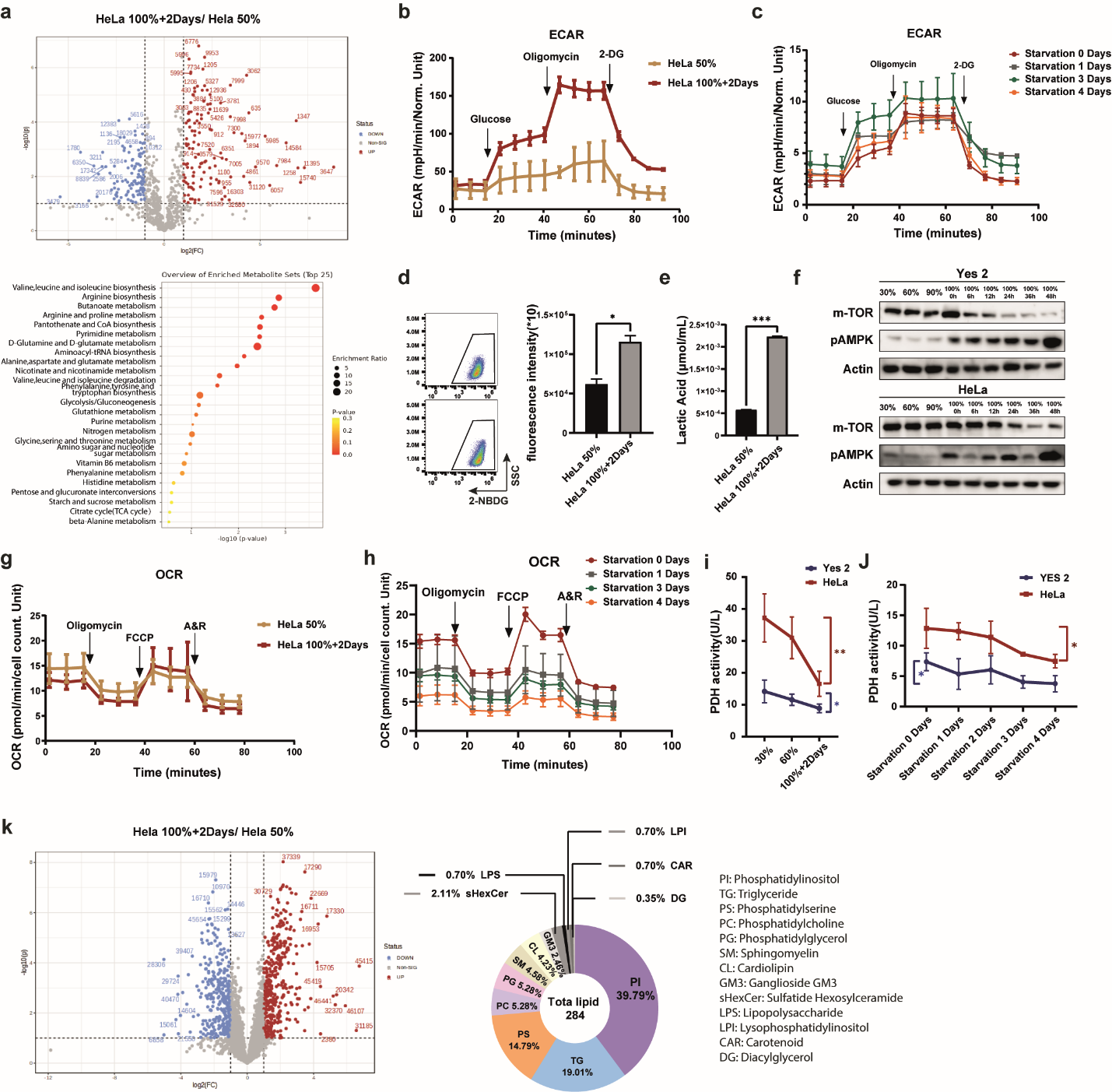
**

Supplementary Fig.6: The differences of metabolomics assay and lipidomic assay of HeLa cells in different cell densities.

**a**, Volcano plot analysis of metabolite by untargeted metabolomics assay. Dotted line(gray): log2(FC)=1, -log10(P)=1.FC: fold change. (Upper) The 95 significantly up-regulated metabolite (fold change>2 and p value<0.05) in the HeLa *CDC25A*-WT 100%+2Days group compared to the HeLa *CDC25A*-WT 50% group (red plots) were enriched in KEGG pathways(down) and the top25 pathways upregulated pathways were showed by p value. **b**, **c** and **g, h** HeLa were seeded in Seahorse XF analyzer culture plates and ECAR in **b, c** and real-time OCR in **g, h**, were measured, with different density treatment in **b, g** and serum starvation treatment in **c, h**. A &R: Antimycin A & Rotenone. **d**, Mean fluorescent intensity (MFI) of 2-NBD-glucose. *p < 0.05. 2-NBDG: 2-NBD-glucose. **e**, Lactic acid assay of HeLa cells in different density. **f**, Western Blot analysis of the expression of m-TOR, pAMPK. **i** and **j**, PDH activity assay of HeLa cells in different density in **i**, and starvation treatment in **j**. *p < 0.05, **p < 0.01. **k,** Volcano plot analysis of metabolite by untargeted lipidomics. Dotted line(gray): log2(FC)=1, -log10(P)=1. FC: fold change (left). The metabolites (red plots) were classified (right, pie chart).

**
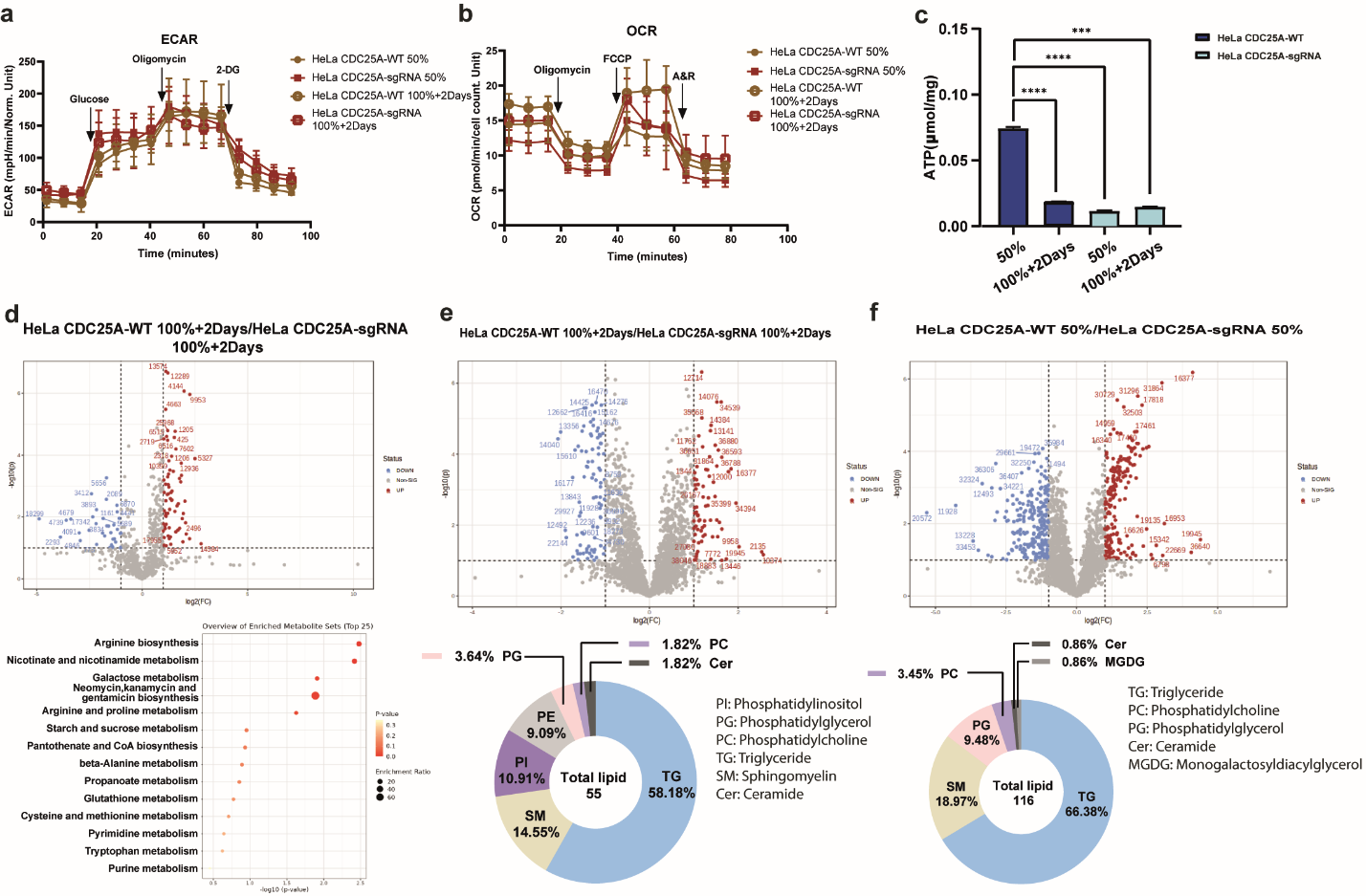
**

Supplementary Fig.7: The differences of metabolomics assay and lipidomic assay between HeLa CDC25A-WT cell line and HeLa CDC25A-sgRNA cell line in different cell densities.

**a-b**, HeLa *CDC25A*-WT and HeLa *CDC25A*-sgRNA cells were seeded in Seahorse XFp analyzer culture plates and ECAR in **a**, and real-time OCR in **b**, were measured, with different density treatment. A&R: Rotenone& Antimycin A. **c**, ATP assay of HeLa *CDC25A*-WT cell line and HeLa *CDC25A*-sgRNA cell line in different density. ***p < 0.001, ****p < 0.0001. **d**, Volcano plot analysis of metabolite by untargeted metabolomics assay. Dotted line(gray): log2(FC)=1, -log10(P)=1.FC: fold change. (Upper) The metabolites (red plots) were enriched in KEGG pathways(down) and the top25 pathways upregulated pathways were showed by P value. **e-f**, Volcano plot analysis of metabolite by untargeted lipidomics. Dotted line(gray): log2(FC)=1, -log10(P)=1. FC: fold change (left). The metabolites (red plots) were classified (right, pie chart).
